# Phosphorylation of EB2 by Aurora B and CDK1 ensures mitotic progression and genome stability

**DOI:** 10.1038/ncomms11117

**Published:** 2016-03-31

**Authors:** Makoto Iimori, Sugiko Watanabe, Shinichi Kiyonari, Kazuaki Matsuoka, Ryo Sakasai, Hiroshi Saeki, Eiji Oki, Hiroyuki Kitao, Yoshihiko Maehara

**Affiliations:** 1 Department of Molecular Oncology, Graduate School of Medical Sciences, Kyushu University, 3-1-1 Maidashi, Higashi-ku, Fukuoka 812-8582, Japan; 2 Innovative Anticancer Strategy for Therapeutics and Diagnosis Group, Innovation Center for Medical Redox Navigation, Kyushu University, 3-1-1 Maidashi, Higashi-ku, Fukuoka 812-8582, Japan; 3 Department of Biochemistry, Nagoya University Graduate School of Medicine, 65 Tsurumai-cho, Showa-ku, Nagoya, Aichi 466-8550, Japan; 4 Taiho Pharmaceutical Co., Ltd., 224-2, Ebisuno, Hiraishi, Kawauchi-cho, Tokushima 771-0194, Japan; 5 Department of Biochemistry I, Kanazawa Medical University, 1-1 Daigaku Uchinada Kahoku, Ishikawa 920-0293, Japan; 6 Department of Surgery and Science, Graduate School of Medical Sciences, Kyushu University, 3-1-1 Maidashi, Higashi-ku, Fukuoka 812-8582, Japan

## Abstract

Temporal regulation of microtubule dynamics is essential for proper progression of mitosis and control of microtubule plus-end tracking proteins by phosphorylation is an essential component of this regulation. Here we show that Aurora B and CDK1 phosphorylate microtubule end-binding protein 2 (EB2) at multiple sites within the amino terminus and a cluster of serine/threonine residues in the linker connecting the calponin homology and end-binding homology domains. EB2 phosphorylation, which is strictly associated with mitotic entry and progression, reduces the binding affinity of EB2 for microtubules. Expression of non-phosphorylatable EB2 induces stable kinetochore microtubule dynamics and delays formation of bipolar metaphase plates in a microtubule binding-dependent manner, and leads to aneuploidy even in unperturbed mitosis. We propose that Aurora B and CDK1 temporally regulate the binding affinity of EB2 for microtubules, thereby ensuring kinetochore microtubule dynamics, proper mitotic progression and genome stability.

## Abstract (web summary)

Temporal regulation of microtubule dynamics in mitosis can be achieved by phosphorylation of microtubule plus-end proteins. Here, the authors show that Aurora B and CDK1 phosphorylate EB2, which changes microtubule binding affinity and controls kinetochore microtubule dynamics and genome stability.

## Graphical abstract

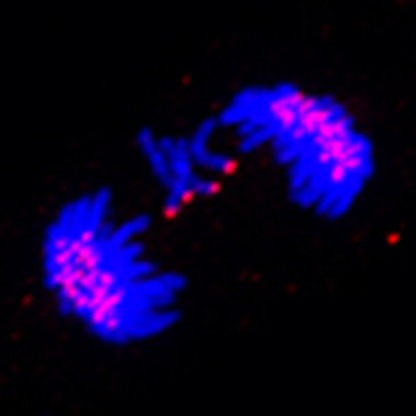

Microtubules (MTs) are highly dynamic polymers that constantly switch between phases of growth and shrinkage^1^,^2^. During mitosis, plus-end dynamics of spindle MTs are required for capture of kinetochores, which ensures proper mitotic progression. Defects in this process can result in genomic instability and aneuploidy, which contribute to tumorigenesis^3^,^4^. In early mitosis, however, many kinetochores engage in incorrect MT attachments. To ensure equal chromosome segregation, Aurora B kinase engages in kinetochore–MT error correction, in particular the destabilization of kinetochore–MT interactions. Reduced turnover of kinetochore–MTs in early mitosis increases the frequency of chromosome malorientation and missegregation^5^. Hence, the temporal regulation of MT dynamics during mitosis is essential for genome stability.

Plus-end tracking proteins (+TIPs), which accumulate selectively at growing MT plus ends, play an important role in regulating the stability of MTs^6^,^7^,^8^,^9^. During mitosis, the activity and localization of many +TIPs are regulated by phosphorylation. For example, phosphorylation of cytoplasmic linker protein (CLIP)-170 by PLK1 and CK2 is essential for kinetochore targeting of CLIP-170 and is involved in the timely formation of kinetochore–MT attachments^10^. CLIP-associating proteins (CLASPs) 1 and 2 associate with kinetochores to promote turnover of attached MTs, to ensure the bipolarity and appropriate size of the mitotic spindle^11^,^12^,^13^,^14^. Specifically, CLASP2 is phosphorylated by CDK1, which primes CLASP2 for association with PLK1, thereby promoting PLK1 recruitment to kinetochores^15^. In addition, the kinesin-13 family member MCAK (also known as Kif2C) is phosphorylated by Aurora B and PLK1, and catalyses MT depolymerization during correction of chromosome malorientation^16^,^17^,^18^,^19^,^20^.

End-binding proteins (EBs) are the most widely conserved family of +TIPs^8^. In mammalian cells, the EB family has three members, EB1, EB2 (RP1) and EB3 (EBF3), each of which is encoded by a different *MAPRE* gene^21^. These proteins contain an N-terminal calponin homology (CH) domain with a highly conserved fold, which is necessary and sufficient for binding to MT plus ends, as well as a coiled-coil region that determines their dimerization^22^,^23^,^24^. The carboxy-terminal region of EBs contains the end-binding homology (EBH) domain, which is important for self-inhibition and binding to various partners^8^. EB1 and EB3 share structural and functional similarities, and influence MT dynamics by promoting growth and preventing catastrophe, whereas EB2 does not^25^,^26^,^27^. During mitosis, EB1 is involved in spindle orientation and stabilization of astral MTs^28^,^29^,^30^,^31^. Furthermore, phosphorylation of EB3 by Aurora B leads to a significant increase in MT growth, resulting in stabilization of the midbody^32^,^33^. However, the mitotic regulation of EB2 is largely unknown.

Here we focus on the regulation of EB2 during mitosis. Aurora B and CDK1 phosphorylate EB2 at multiple sites, thereby reducing its binding affinity for MTs. We provide the first evidence that the phosphoregulation of EB2 is required for proper mitotic progression and discuss the spatiotemporal regulation of EB2 in light of the previously demonstrated Aurora B phosphorylation gradient and its contribution to genome stability.

## Results

### EB2 is phosphorylated by CDK1 during mitosis

**Figure 1:**
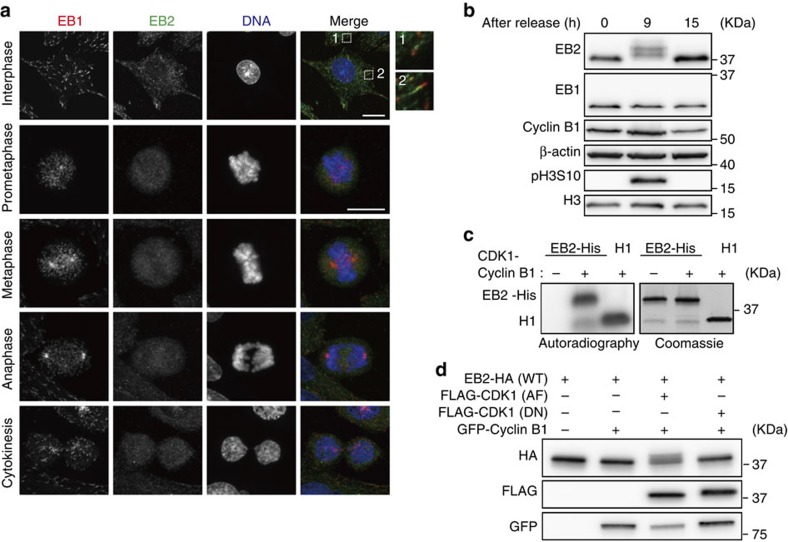
EB2 is phosphorylated and not localized along MTs during mitosis. (**a**) Representative confocal images showing the localization of EB1 and EB2 throughout the cell cycle. Cells were co-stained with antibodies against EB1 (red), EB2 (green) and DAPI (blue). Scale bars, 5 μm. (**b**) HeLa cells were synchronized by a double-thymidine block and released into fresh medium. See Supplementary Fig. 9a for experimental scheme. Immunoblot analysis was carried out using antibodies against the indicated proteins. (**c**) *In vitro* CDK1 kinase assay. Left, active CDK1–Cyclin B1 was incubated with the indicated recombinant proteins in the presence of [γ-^32^P]-ATP. Right, the total amount of recombinant proteins from the same reaction were resolved by SDS–PAGE and Coomassie Brilliant Blue (CBB) staining. (**d**) HEK293T cells were co-transfected with EB2-HA in combination with GFP–Cyclin B1 and constitutively active FLAG-CDK1 (AF) or kinase-dead FLAG-CDK1 (DN), and incubated for 24 h. Immunoblot analysis was carried out using antibodies against the indicated tags.

Consistent with previous work, we found that EB2 in interphase cells was evenly distributed along MT lattices and exhibited only a very slight accumulation at the plus ends of MTs (Fig. 1a and see ref. 25). In mitotic cells, on the other hand, EB2 was dispersed rather than localized along MT lattices (Fig. 1a). By contrast, EB1 was clearly localized at the plus ends of spindle MTs and centrosomes throughout mitosis. Hence, we investigated the possibility that the level of EB2 is substantially reduced during mitosis. Although the protein level of EB2 was decreased by ∼35% at 9–12 h after the release from double-thymidine block, when most cells were in mitosis, we observed no dramatic reduction in EB2 (Supplementary Fig. 1a). Here we noticed that the bands of EB2 proteins observed in normal SDS–polyacrylamide gel electrophoresis (PAGE) gels were only slightly shifted during mitosis. Therefore, we then performed phosphate-affinity SDS–PAGE using Phos-tag gel, in which phosphorylated proteins are mobility shifted, followed by immunoblotting. In this experiment, EB2 proteins were detected as hypershifted bands, suggesting that EB2 was phosphorylated during mitosis (Supplementary Fig. 1a). We confirmed the mitotic phosphorylation of EB2 in highly synchronous mitotic cells, prepared by mitotic shake-off after double-thymidine block and release (Fig. 1b). The upper bands of EB2 were diminished after phosphatase treatment, indicating that EB2 was indeed phosphorylated at multiple sites (Supplementary Fig. 1b,c left). In addition, a doublet band of EB2 proteins observed at each time point in the Phos-tag gel after double-thymidine block and release was also diminished after phosphatase treatment, indicating that EB2 was phosphorylated at a single site throughout the cell cycle (Supplementary Fig. 1a,c right).

As CDK1 kinase is a key regulator of mitosis, we speculated that CDK1 might phosphorylate EB2. To investigate this possibility, we subjected EB2 to *in vitro* kinase assays in the presence or absence of CDK1-Cyclin B1. Indeed, CDK1 phosphorylated recombinant EB2 (Fig. 1c). Consistent with this observation, overexpression of constitutively active CDK1 (Y14A, Y15F (AF))^34^, but not the kinase-dead mutant (D145N (DN))^35^, led to a mobility shift of EB2-HA (Fig. 1d). Taken together, our results suggest that EB2 is phosphorylated by CDK1 during mitosis.

### Phosphorylation of EB2 regulates the binding affinity to MTs

**Figure 2:**
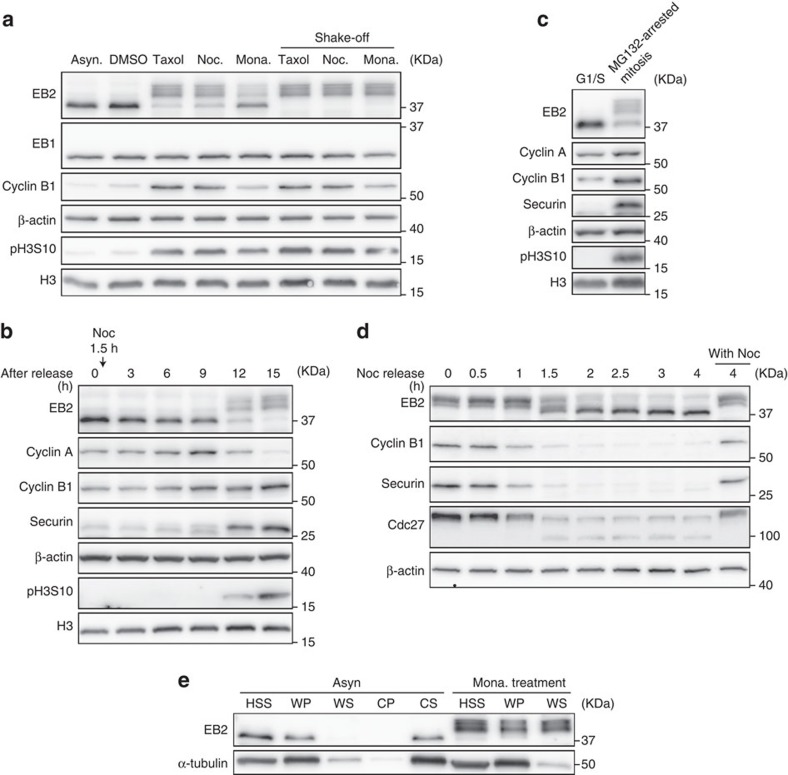
Hyperphosphorylation of EB2 during SAC-dependent arrest. (**a**) Immunoblot analysis of EB2 in asynchronous (Asyn.), taxol-arrested, nocodazole-arrested (Noc.) and monastrol-arrested (Mona.) extracts from whole or shake-off HeLa cells. (**b**,**c**) HeLa cells were synchronized by a double-thymidine block and then nocodazole was added 1.5 h after release (**b**), or MG132 was added 6.5 h after release (**c**). See Supplementary Fig. 9b for experimental scheme. (**d**) HeLa cells were synchronized by a single thymidine block and then after 3 h released into nocodazole for 11 h. Synchronized HeLa cells were harvested by mitotic shake-off and transferred into fresh medium with or without nocodazole. See Supplementary Fig. 9c for experimental scheme. (**e**) MT co-sedimentation assay. Asynchronous and monastrol-arrested HeLa cells were lysed, pre-cleared (HSS) and subjected to a MT co-sedimentation assay. HSS was incubated for 30 min with exogenous purified porcine brain tubulin that was either taxol-stabilized at 37 °C (WP and WS fractions) or colchicine-depolymerized on ice (CP and CS fractions). The samples were centrifuged at 100,000 *g* to separate the MT polymers (WP and CP) from the soluble tubulin dimers (WS and CS) and immunoblot analysis was carried out to assess the presence of EB2 and tubulin. See Supplementary Fig. 9d for experimental scheme. CP, cold pellet; CS, cold supernatant; HSS, high-speed supernatant; WP, warm pellet; WS, warm supernatant. Immunoblot analysis was carried out using antibodies against the indicated proteins.

To investigate mitotic EB2 in detail, we treated various human cell lines with drugs that interfere with mitosis (hereafter, ‘mitotic drugs') such as taxol, nocodazole and monastrol. Under these conditions, EB2 was detected in immunoblotting experiments as hyperphosphorylated bands in HeLa cells (Fig. 2a) and several cell lines (Supplementary Fig. 2a–e). This hyperphosphorylation was also induced by docetaxel, vinblastine, vincristine and eribulin, cancer chemotherapeutic drugs that target MTs (Supplementary Fig. 2f). Here, hyperphosphorylated bands of EB2 were weaker in monastrol-arrested extracts of whole HeLa cells than in other mitotic drug-arrested extracts. We attribute this weaker phosphorylation level to the mitotic index, because the cyclin B1 level is low in monastrol-treated cells and the hyperphosphorylated EB2 level does not vary among highly synchronous mitotic cells treated with various mitotic drugs. We next investigated whether hyperphosphorylation of EB2 was associated with mitotic entry and arrest. To this end, we monitored phosphorylation of EB2 in synchronized HeLa cells arrested by the MT drugs nocodazole (Fig. 2b), taxol (Supplementary Fig. 3) or the non-MT-targeting drug MG132, which blocks APC-dependent proteolysis, leading to accumulation of securin and cyclin B1, and spindle assembly checkpoint (SAC)-independent mitotic arrest (Fig. 2c). EB2 hyperphosphorylation was induced in MT drug-arrested prometaphase cells and in MG132-arrested metaphase cells, consistent with the accumulation of securin and phospho-histone H3 Ser10. In addition, the kinetics of EB2 dephosphorylation were similar to those of Cdc27, a known CDK1 substrate, and consistent with the kinetics of degradation of securin and cyclin B1 (Fig. 2d). These results suggest that EB2 is hyperphosphorylated during early to mid-mitosis and is dephosphorylated during mitotic exit.

Next, we investigated the functional significance of EB2 phosphorylation. Previous reports demonstrated that multi-site phosphorylation of Mal3 or Bim1p, the fission or budding yeast homologues of EB1, reduces their affinity to MTs^36^,^37^. Another study showed that removal of the negatively charged C-terminal domain from EB1 results in stable association with the MT lattice, suggesting that electrostatic repulsion prevents stable binding of the wild-type (WT) protein^38^. On the basis of these earlier findings, we hypothesized that phosphorylation of EB2 regulates this protein's affinity for MTs during mitosis. To test this idea, we examined the contribution of EB2 phosphorylation to MT binding using a MT co-sedimentation assay. EB2 proteins in asynchronous HeLa cells were enriched in the polymerized MT pellet fraction (warm pellet (WP)), but not the soluble tubulin fraction (warm supernatant (WS)), whereas EB2 proteins were present in the soluble tubulin fraction prepared by preventing MT polymerization (cold supernatant (CS)) (Fig. 2e). This finding suggests that non-phosphorylated EB2 binds polymerized MT rather than soluble tubulin. By contrast, hyperphosphorylated EB2, which was extracted from monastrol-arrested prometaphase cells to avoid carryover of the MT drugs, was detected in both the polymerized MT pellet fraction (WP) and the soluble tubulin fraction (WS) (Fig. 2e). Thus, we conclude that hyperphosphorylation of EB2 reduces the protein's binding affinity for MTs.

### Aurora B phosphorylates EB2

**Figure 3:**
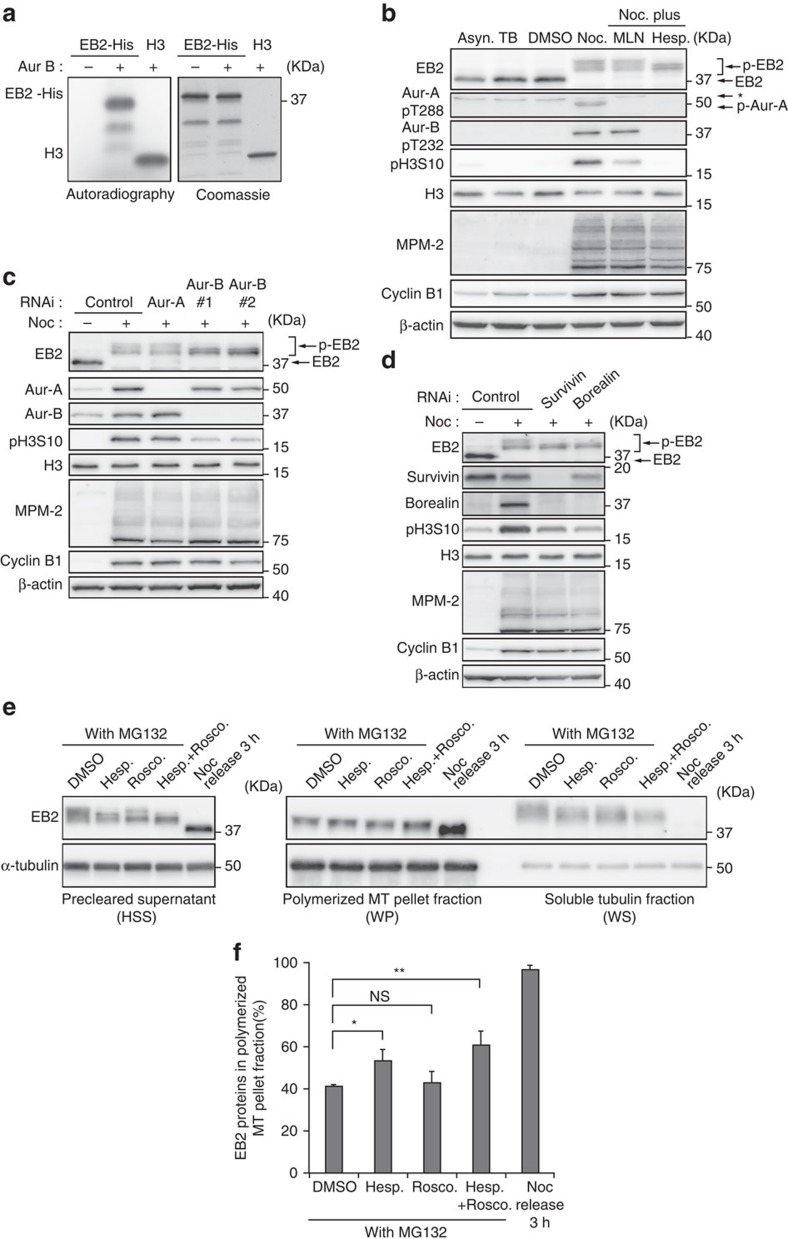
Aurora B phosphorylates EB2. (**a**) *In vitro* Aurora B kinase assay. Left, active Aurora B was incubated with recombinant proteins as indicated in the presence of [γ-^32^P]-ATP. Right, the total amount of recombinant proteins from the same reaction were resolved by SDS–PAGE and stained with Coomassie Brilliant Blue (CBB). (**b**) HeLa cells were synchronized by a double-thymidine block; after 6.5 h, nocodazole was added with or without MLN8054 (Aurora A inhibitor) or hesperadin (Aurora B inhibitor). See Supplementary Fig. 9e for experimental scheme. Mitotic cells were harvested by mitotic shake-off. Levels of MPM-2 and Cyclin B1 were determined as a mitosis-specific marker. (**c**,**d**) HeLa cells were transfected with siRNAs as indicated; after 24 h, the cells were treated with nocodazole for 4 h. Mitotic cells were harvested by mitotic shake-off. (**e**) HeLa cells were synchronized by a single thymidine block and then after 3 h released into nocodazole for 11 h. Synchronized mitotic HeLa cells were harvested by mitotic shake-off, washed and released into MG132 with or without roscovitine (CDK1 inhibitor) and/or hesperadin for 3 h. See Supplementary Fig. 9f for experimental scheme. (**f**) HeLa cells were harvested as shown in **e**, lysed, pre-cleared (high-speed supernatant (HSS)) and subjected to a MT co-sedimentation assay. HSS was incubated for 30 min with exogenous purified porcine brain tubulin that was taxol-stabilized at 37 °C. The sample was centrifuged at 100,000 *g* to separate MT polymers (WP) from soluble tubulin dimers (WS) and immunoblot analysis was carried out to assess the presence of EB2. The distribution of EB2 in the WP fraction was calculated based on the intensity of each band, as assessed by densitometry. Data are means± s.d. from three independent experiments. *P<0.05 and **P<0.01, NS, not significant (unpaired *t*-test) compared with control (dimethyl sulfoxide (DMSO)).

As indicated above, phosphorylated EB2 was detected as hypershifted bands in highly synchronous mitotic cells and in cells treated with mitotic drugs, suggesting that EB2 was phosphorylated at multiple sites (Figs 1b and 2a). Hence, we hypothesized that other kinases could phosphorylate EB2, in addition to CDK1 (Fig. 1c,d). Previous studies showed that Aurora B phosphorylates EB3 and the budding yeast EB1 homologue Bim1p during anaphase and cytokinesis^32^,^33^,^37^. In addition, Aurora B phosphorylates recombinant EB2 (GST-EB2) *in vitro*^33^. To explore the possibility that EB2 is a substrate of Aurora B, we subjected EB2 to an *in vitro* kinase assay in the presence or absence of Aurora B, and found that the kinase phosphorylated recombinant EB2 (EB2-His) (Fig. 3a). Furthermore, to determine whether EB2 is phosphorylated by Aurora B in cells, we synchronized HeLa cells by thymidine–nocodazole arrest, with or without Aurora inhibitors, and then isolated mitotic cells by shake-off. Hyperphosphorylation of EB2 was partially abolished in the presence of hesperadin, an Aurora B kinase inhibitor^39^, but not in the presence of MLN8054, an Aurora A kinase inhibitor^40^ (Fig. 3b). Consistent with this observation, hyperphosphorylation of EB2 was partially diminished in Aurora B-knockdown cells but not in Aurora A-knockdown cells (Fig. 3c). Aurora B is a component of chromosomal passenger complex, which consists of the kinase and three non-enzymatic subunits, INCENP, survivin and borealin, which regulate the localization, enzymatic activity and stability of Aurora B kinase^41^. To confirm the contribution of Aurora B (as a component of chromosomal passenger complex) to EB2 phosphorylation, we monitored phosphorylation levels in survivin- or borealin-knockdown cells, yielding results similar to those obtained in Aurora B-knockdown cells (Fig. 3c,d). Collectively, these results demonstrate that Aurora B phosphorylates EB2 during SAC-dependent arrest in cells.

### Aurora B and CDK1 maintain the phosphorylation of EB2

As shown in Fig. 2a–c, hyperphosphorylation of EB2 appears to be triggered by mitotic entry and SAC activation. We attempted to determine whether hyperphosphorylation of EB2 was maintained while SAC was activated. To test this, we released HeLa cells from thymidine–nocodazole arrest into MG132-containing medium. The hyperphosphorylation of EB2 was maintained 3 h after the release from nocodazole into MG132; at that time, in the absence of MG132, most cells had exited from mitosis (Supplementary Fig. 4). Previous studies showed that disassembly of the mitotic checkpoint complex, a critical inhibitor of APC/C, is blocked in MG132-arrested metaphase cells, suggesting that the SAC is not inactivated in these cells^42^,^43^,^44^,^45^. Therefore, hyperphosphorylation of EB2 is maintained while SAC is activated. In addition, we found that inhibition of Aurora B or CDK1 by hesperadin or roscovitine, respectively, partially abolished the hyperphosphorylation of EB2 in arrested metaphase cells following nocodazole block and release into MG132; moreover, simultaneous inhibition both of Aurora B and CDK1 had an additive effect on hyperphosphorylation of EB2 (Supplementary Fig. 4). Consistent with these observations, EB2 protein levels in the polymerized MT pellet fraction (WP) were significantly elevated when Aurora B was inhibited, or both Aurora B and CDK1 were simultaneously inhibited, but not when CDK1 was inhibited alone (Fig. 3e,f). Together, these data indicate that Aurora B and CDK1 phosphorylate EB2 at distinct sites and maintain the hyperphosphorylation of EB2 and its binding affinity to MTs while SAC activity is sustained.

### EB2 phosphorylation does not depend on its localization

**Figure 4:**
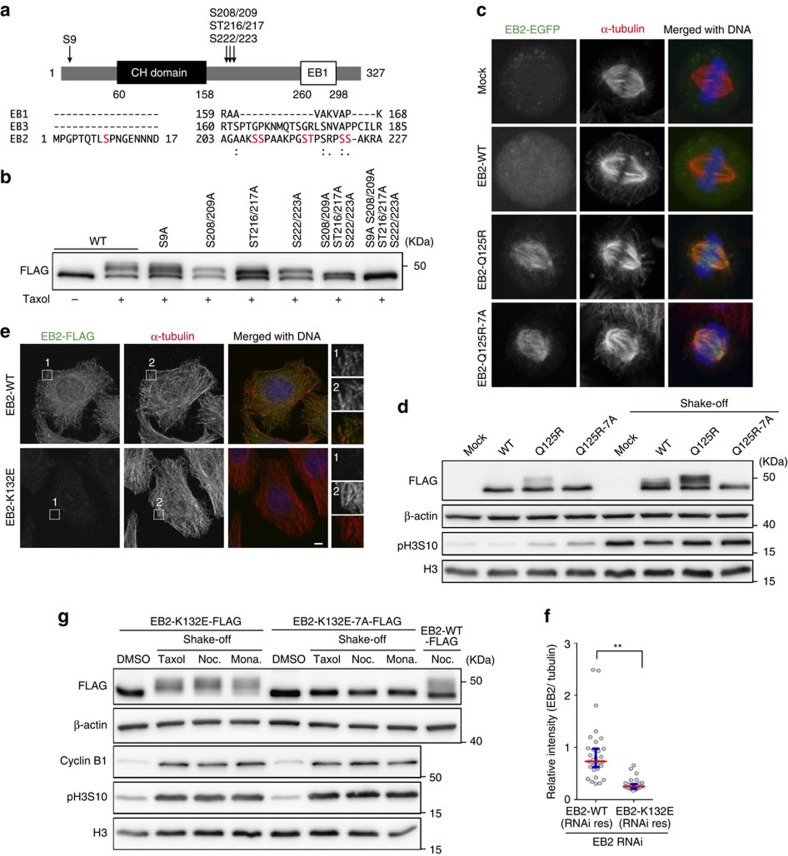
Phosphorylation level of EB2 does not depend on its subcellular localization. (**a**) Identification of EB2 phosphorylation sites. Arrows indicate the major phosphorylation sites, which were identified on the basis of alanine substitution in the Aurora B and CDK1 consensus sequences. Alanine-substitution sites of EB2 are shown with red in multiple sequence alignments of human EBs. (**b**) Cell extracts from HeLa cells transiently expressing EB2-FLAG WT or its phospho-site mutants in the presence of taxol were immunoblotted with an anti-FLAG antibody. (**c**) HeLa cells expressing EB2-EGFP WT, a MT-targeted mutant (EB2-Q125R), or its non-phosphorylatable double mutant (EB2-Q125R-7A) were fixed and stained with anti-tubulin antibody and DAPI. (**d**) Immunoblot analysis of EB2 in extracts from asynchronous or mitotic (shake-off) HeLa cells expressing EB2-EGFP WT, EB2-Q125R mutant or EB2-Q125R-7A double mutant. (**e**,**f**) Immunofluorescence images of HeLa Flp-In cells expressing EB2-FLAG WT (EB2-WT) or MT-binding site mutant (EB2-K132E). Cells were transfected with siRNA against endogenous EB2, incubated for 48 h, fixed and co-stained with anti-tubulin and anti-FLAG antibodies. Fluorescence intensities of EB2 were quantified in **f**. Open circles represents individual cells (*n*≥34) from three independent experiments. Red lines, medians; blue bars, interquartile range. **P<0.0001, NS, not significant (Mann–Whitney *U*-test). (**g**) Immunoblot analysis of EB2 in asynchronous (dimethyl sulfoxide (DMSO)), taxol-arrested, nocodazole-arrested (Noc.) and monastrol-arrested (Mona.) extracts from mitotic (shake-off) HeLa Flp-In cells, expressing EB2-FLAG MT-binding site mutant (EB2-K132E) or double mutant (EB2-K132E-7A). Cells were transfected with siRNA against endogenous EB2 and then incubated for 48 h before lysis. Scale bars, 5 μm.

To determine the biological significance of EB2 phosphorylation, we first attempted to identify the phosphorylation sites in EB2. EB2 has five consensus sequences for Aurora B phosphorylation ([R/K]X[S/T]) and four for CDK1 ([S/T]P). To determine the phosphorylation sites, we replaced serine/threonine residues with alanine and found that replacement of any one of four serines (S9, S208/209, ST216/217 and S222/223) effectively suppressed the phosphorylation at each individual residue (Fig. 4a,b). Furthermore, alanine substitutions of all four sites completely abolished the hyperphosphorylation of EB2 (Fig. 4b).

We next attempted to determine whether EB2 was phosphorylated while binding to spindle MTs. A previously identified point mutation (Q89R or Q89A) in the CH domain of Mal3, the fission yeast homologue of EB1, increases the protein's binding affinity for MT lattices^36^,^46^. Therefore, we constructed the analogous mutant (Q125R) in human EB2 and expressed it in HeLa cells. In contrast to EB2-WT, EB2-Q125R apparently localized along spindle MTs (Fig. 4c). When EB2-Q125R was forced to be localized to spindle MTs, the protein was hyperphosphorylated, whereas a non-phosphorylatable EB2-Q125R mutant (EB2-Q125R-7A) was not (Fig. 4c,d). These data suggest that EB2 is phosphorylated while binding to spindle MTs.

Next, we investigated whether EB2 is phosphorylated in the cytoplasm. To this end, we established a cell line that stably expresses an RNA interference (RNAi)-resistant EB2 mutant that disrupts MT binding. Previous work showed that Lys-89 in EB1 is essential for binding to MTs *in vitro*^24^ and is highly conserved among human EBs. Hence, we generated a mutant in which Lys-132 of EB2, corresponding to Lys-89 of EB1, was replaced with glutamate (EB2-K132E). As expected, EB2-K132E proteins did not localize along MT lattices (Fig. 4e,f). Even when not localized to spindle MTs, however, EB2-K132E proteins were phosphorylated following treatment with mitotic inhibitors, whereas non-phosphorylatable EB2-K132E mutant (EB2-K132E-7A) proteins were not (Fig. 4g). Notably, the phosphorylation level of EB2-K132E was nearly identical to that of EB2-WT (Fig. 4g). Our data show that EB2 is phosphorylated not only while binding to spindle MTs but also in the cytoplasm, and that the phosphorylation levels of MT-bound and cytoplasmic EB2 were not significantly different.

### Phosphorylation of EB2 is required for proper mitosis

**Figure 5:**
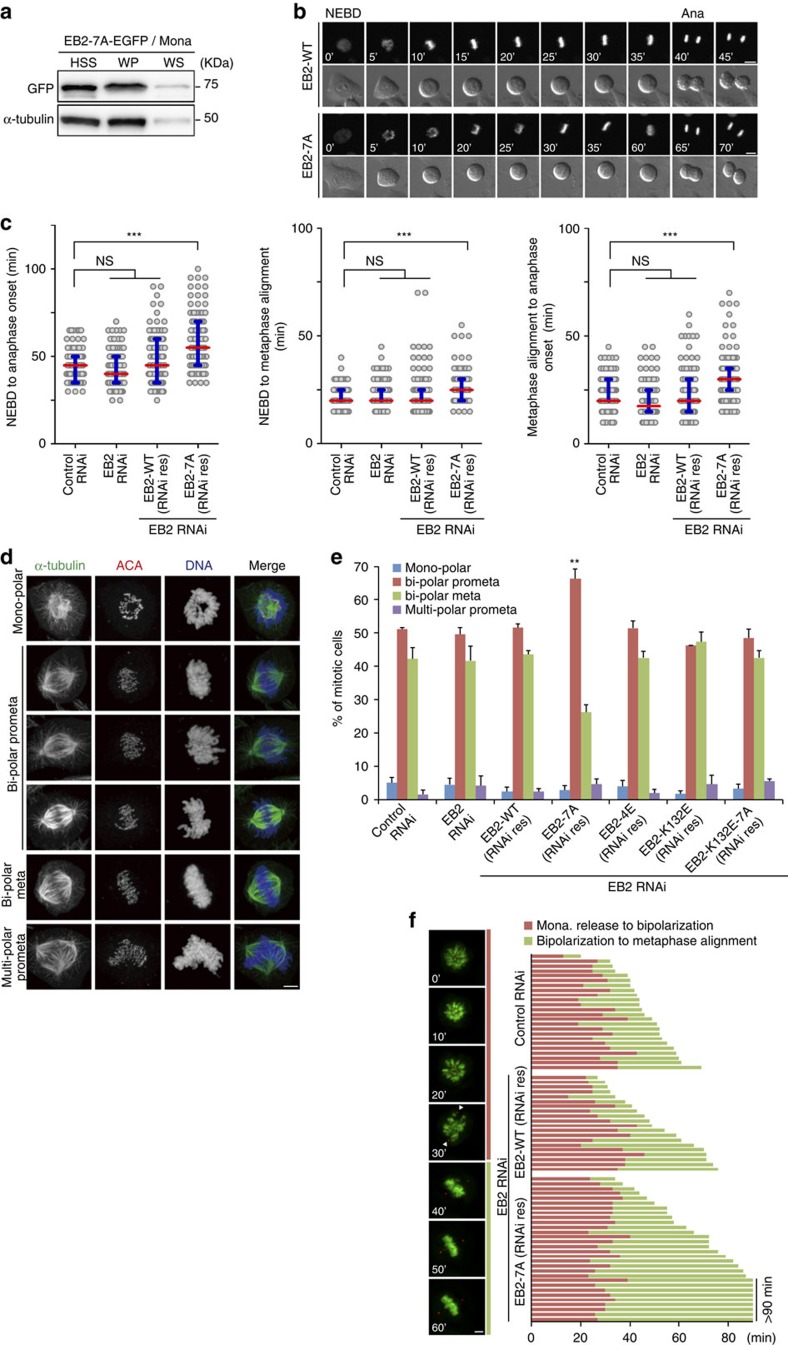
Effect of non-phosphorylatable EB2 on mitotic progression. (**a**) HeLa Flp-In cells stably expressing RNAi-resistant non-phosphorylatable EB2-EGFP (EB2-7A-EGFP) were transfected with siRNA against endogenous EB2 and treated with monastrol. A MT co-sedimentation assay was performed to separate MT polymers (WP) from soluble tubulin dimers (WS). Immunoblot analysis was performed to assess the presence of EB2-7A-EGFP and tubulin. (**b**) Selected frames from live-cell imaging of representative EB2-FLAG WT (EB2-WT)- or non-phosphorylatable mutant (EB2-7A)-expressing cells expressing histone H2B-mCherry. Time (minutes) after NEBD is shown on the images. Ana, onset of anaphase. (**c**) Live-cell images were analysed from NEBD to Ana in EB2 knockdown, EB2-WT or -7A and control knockdown cells (*n*≥100) from three independent experiments. Open circles represent individual cells. Red lines, median; blue bars, interquartile range. ***P<0.0001; NS, not significant (Mann-Whitney *U*-test). (**d**,**e**) Monastrol washout experiment. HeLa Flp-In cells stably expressing RNAi-resistant EB2-FLAG WT (EB2-WT), non-phosphorylatable mutant (EB2-7A), phospho-mimetic mutant (EB2-4E), MT-binding site mutant (EB2-K132E) or its non-phosphorylatable mutant (EB2-K132E-7A) were transfected with siRNA against endogenous EB2. Cells were treated with monastrol for 2 h, transferred into fresh medium with MG132 for 1 h and then fixed and co-stained with anti-tubulin antibody, ACA and DAPI. Representative images are shown in **d**. Data are means± s.d. from three independent experiments (≥250 cells per experiment). **P<0.001 (unpaired *t*-test) compared with control RNAi. (**f**) Left, selected frames from live-cell imaging of representative cells expressing histone H2B-GFP and pericentrin-RFP. The amount of time (minutes) after monastrol washout is shown on the images. The timing of bipolar spindle assembly was measured by the maximum pole-to-pole distance (arrowheads highlight the centrosome position). Right, graph of the cumulative duration of metaphase alignment after monastrol washout. Bars indicate the time from monastrol washout to bipolar spindle assembly (red) and from the first frame of bipolar spindle assembly to metaphase alignment (green) in control (*n*=24), EB2-WT-expressing (*n*=21) and EB2-7A-expressing cells (*n*=30) from three independent experiments. Each bar represents one cell. Scale bars, 5 μm.

To confirm that phosphorylation of EB2 is indispensable for dissociation from MTs and subsequent mitotic progression, we established HeLa Flp-In cell lines stably expressing an RNAi-resistant EB2 (Supplementary Fig. 5a). A previous study and our data showed that overexpression of EB1 leads to MT bundling (Supplementary Fig. 5b,d and see ref. 47). Thus, we assessed the effects of overexpression of EB2 on MT bundling and arrays, and confirmed that overexpression of EB2 did not have an effect, at least when the overexpression level was sixfold higher than the endogenous EB2 level (Supplementary Fig. 5c,d). We then performed MT co-sedimentation assay and found that the non-phosphorylatable EB2 (EB2-7A) preferentially bound to MTs *in vitro*, as revealed by its presence in the polymerized MT pellet fraction (WP) (Fig. 5a). These results suggest that mitotic EB2-7A proteins can potentially bind to MTs.

We next examined mitotic progression in asynchronous EB2-7A cells. We measured the duration of mitosis in HeLa cells stably expressing EB2-WT or EB2-7A along with mCherry-tagged histone H2B. Live-cell imaging analyses verified that the interval of time from nuclear envelope breakdown (NEBD) to onset of anaphase was significantly lengthened in EB2-7A cells (Fig. 5b,c left). Furthermore, the prolonged mitosis in EB2-7A-expressing cells was caused not only by lengthening of the duration from NEBD to metaphase alignment but also in that from metaphase alignment to anaphase onset (Fig. 5c middle and right). These results suggest that expression of EB2-7A caused significant mitotic delay during an unperturbed cell cycle.

To evaluate the influence of EB2-7A on the response to release from SAC-dependent arrest, we performed a monastrol washout experiment. Treatment of mitotic cells with monastrol causes accumulation of cells with monopolar spindles (Supplementary Fig. 6a and see ref. 48), resulting in the formation of numerous syntelically mis-attached sister kinetochores^49^. As monastrol is a reversible inhibitor, monopolar spindles are converted to bipolar spindles following washout, allowing the correction of erroneous attachments to amphitelic attachments^49^,^50^. Relative to controls, EB2-7A cells exhibited elevated formation of bipolar prometaphases, but not monopolar spindle formation, after monastrol washout (Fig. 5d,e). This observation suggests that the cells had undergone a delay in mitotic progression, but that this event did not influence error correction of erroneous attached chromosomes. Here we note that the efficiency of the formation of bipolar metaphase plates after monastrol washout was 20% lower in Flp-In HeLa cells than in the other HeLa cell lines (Supplementary Fig. 6b). However, the Flp-In system guarantees that the phenotypic differences observed in the different EB2 mutant cell lines are not a result of the variation in the genetic background of the integration site and this is a significant advantage for comparative analysis among different EB2 mutant-expressing cell lines.

In addition, we replaced the serine/threonine residues at each of four phosphorylation sites (S9, S209, T217 and S223) with glutamate, an amino acid that mimics phosphorylation (Supplementary Fig. 5a right end), because each serine/threonine residue at four sites within the serine/serine or serine/threonine tandem motifs (S9, S208/209, ST216/217 and S222/223) was especially important for effective suppression of EB2 hyperphosphorylation (Supplementary Fig. 5e). In the monastrol washout experiment, cells expressing phospho-mimetic EB2 (EB2-4E) exhibited phenotypes similar to those of control cells (Fig. 5e). To carry out detailed analysis of a delay in mitotic progression, we performed live-cell imaging of cells expressing histone H2B-GFP and pericentrin-red fluorescent protein (RFP) during monastrol washout, and compared the efficiency of spindle bipolarization and chromosome alignment (Fig. 5f left and Supplementary Movie 1). EB2-7A expression-induced mitotic delay was due to an increase in the duration of chromosome alignment, whereas the duration from monastrol release to spindle bipolarization was almost unchanged relative to control cells (Fig. 5f right). Together, our data support a model in which hyperphosphorylation of EB2 is required for proper mitotic progression following recovery from mitotic drug-induced SAC-dependent arrest.

In relation to the role of Aurora B in mitotic error correction, previous studies showed that phosphorylation of Hec1 (also known as NDC80), a component of the NDC80 complex, by Aurora B strongly destabilizes kinetochore–MT attachments, an important aspect of the error correction mechanism^51^,^52^,^53^. To investigate whether EB2 is also involved in this pathway, we quantified Hec1 phosphorylation at kinetochores in EB2-WT and EB2-7A cells. We observed no significant changes in Hec1 phosphorylation, or the total amount of Hec1, between EB2-WT and EB2-7A cells (Supplementary Fig. 7a,b), indicating that the non-phosphorylatable EB2 did not affect Hec1 phosphorylation. To confirm that expression of EB2-7A does not affect the number of erroneous attachments, we measured the interkinetochore distance after monastrol washout in control, EB2-WT-expressing and EB2-7A-expressing cells during prometaphase and metaphase. We found no significant changes in the interkinetochore distance among these cells, whereas the distance was decreased in cells released into media containing hesperadin, which inhibits Aurora B and leads to the formation of syntelically erroneous kinetochore–MT attachments (Supplementary Fig. 7c). These observations suggest that hyperphosphorylation of EB2 is required by Aurora B, independently of Hec1 phosphorylation, to ensure proper mitotic progression.

Next, we investigated whether the delay in mitotic progression in EB2-7A cells depends on binding to MTs. In a monastrol washout experiment, cells expressing EB2-K132E-7A, a mutant that displays both abrogated phosphorylation and an abrogated binding affinity for MTs, did not exhibit increased formation of bipolar prometaphases, suggesting that the delay in mitotic progression in EB2-7A-expressing cells depends on binding of EB2-7A to MTs (Figs 4g and 5e). We concluded that delayed mitotic progression in non-phosphorylatable EB2 cells depends on binding to MTs, and that phosphorylation-dependent dissociation of EB2 from MTs is important for proper mitotic progression.

### Phosphorylation of EB2 ensures kinetochore MT dynamics

**Figure 6:**
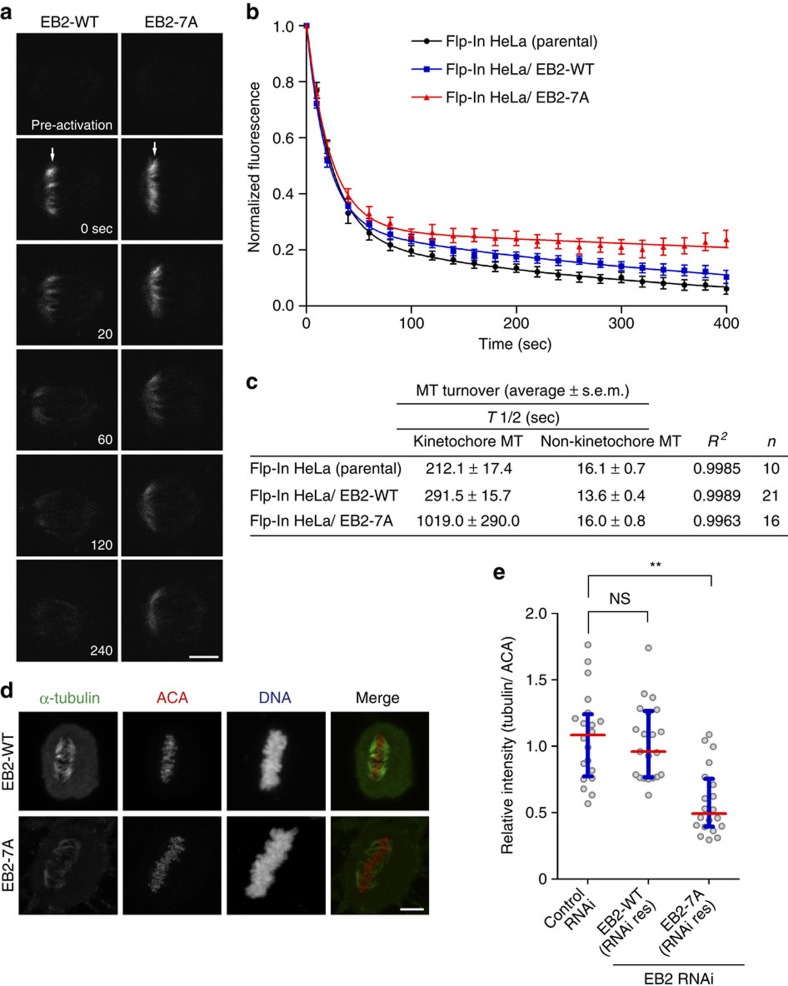
Effect of non-phosphorylatable EB2 on kinetochore–MT stability. (**a**) Selected frames from live-cell imaging of representative EB2-WT- or EB2-7A-expressing cells expressing photoactivatable GFP–tubulin before and after photoactivation. The arrow indicates the activated region. (**b**) Normalized fluorescence intensity of the activated region in control cells (black), EB2-WT-expressing cells (blue) and EB2-7A-expressing cells (red). Data are means±s.e.m. (≥10 cells from three independent experiments). (**c**) Summary of MT turnover measurements in **b**. (**d**,**e**) Immunofluorescence images of cold-stable MTs in metaphase HeLa Flp-In control cells, EB2-WT-expressing cells and EB2-7A-expressing cells. Cells were transfected with siRNA against endogenous EB2 and incubated for 48 h. After transfection, cells were exposed to cold treatment, fixed and co-stained with an anti-tubulin antibody, ACA and DAPI. The fluorescence intensities of tubulin were quantified in **e**. Open circles represents individual cells (*n*≥20) from three independent experiments. Red lines, medians; blue bars, interquartile range. **P<0.0001; NS, not significant (Mann-Whitney *U*-test). Scale bars, 5 μm.

To examine whether non-phosphorylatable EB2 directly affects kinetochore MT dynamics, we measured MT turnover in EB2-7A-expressing cells stably expressing photoactivatable green fluorescent protein (GFP)–tubulin (Fig. 6a–c). In late prometaphase, the half-life of kinetochore MTs was longer in EB2-7A-expressing cells than in control and EB2-WT-expressing cells, whereas the non-kinetochore MT was not (Fig. 6c), suggesting that non-phosphorylatable EB2 contributes to the induction of stable kinetochore MT dynamics.

EB2-7A expression-induced mitotic delay was caused not only by a delay in prometaphase but also by lengthening of the duration from metaphase alignment to anaphase onset (Fig. 5c). This observation may be attributable to a delay in the assembly of stable kinetochore–MT attachments (also known as end-on attachments). Therefore, we performed cold-induced MT depolymerization and quantified the fluorescence of the remaining MTs, to determine the effect on the stable kinetochore–MT attachments in unchallenged metaphase cells. The fluorescence intensity of the remaining MTs after cold treatment was significantly decreased by ∼50% in metaphase EB2-7A-expressing cells in comparison with control and EB2-WT-expressing cells, suggesting a delay in the assembly of stable kinetochore–MT attachments (Fig. 6d,e). Here, in contrast to unchallenged metaphase cells, the assembly of stable kinetochore–MT attachments did not differ between MG132 (a proteasome inhibitor)-arrested metaphase cells expressing EB2-WT and those expressing EB2-7A (Supplementary Fig. 7c), with MG132 seeming to provide extra time for error correction by prolonging metaphase as shown previously^54^,^55^. Together, these data indicate that phosphorylation of EB2 is important in kinetochore MT dynamics and the stability of attachments.

### Non-phosphorylatable EB2 causes chromosomal instability

**Figure 7:**
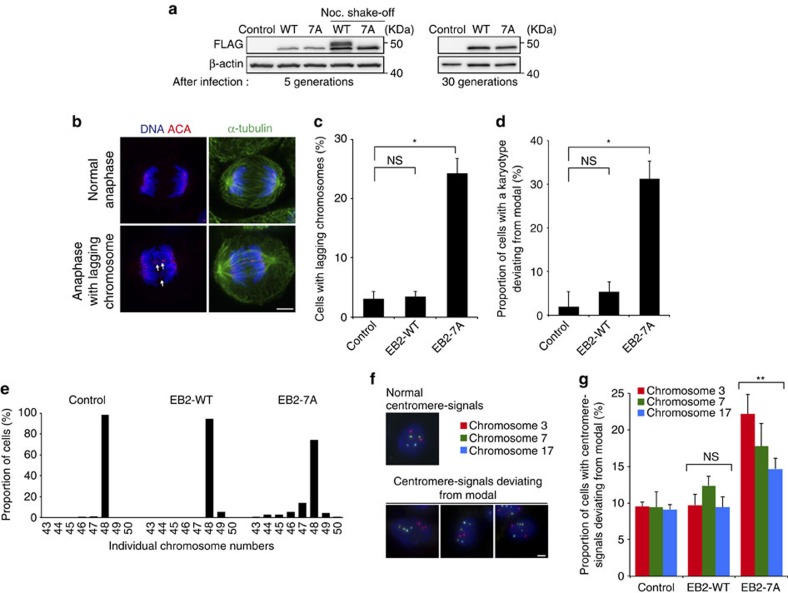
Expression of non-phosphorylatable EB2 leads to lagging chromosomes and aneuploidy. (**a**) MCF10A cells were infected to generate a polyclonal population expressing FLAG-tagged EB2-WT and EB2-7A. Cell extracts were immunoblotted with the indicated antibodies. (**b**,**c**) Quantification of the proportions of EB2-WT- and EB2-7A-expressing MCF10A cells exhibiting lagging chromosomes. Representative images of normal and lagging chromosomes in **b**. Arrows indicate lagging chromosomes. Data are means± s.d. from three independent experiments (≥200 cells per experiment). *P<0.001; NS, not significant (unpaired *t*-test). (**d**,**e**) Proportion of cells with karyotypes deviating from the modal chromosome number was determined after 30 generations in the indicated cells. Individual chromosome numbers from chromosome spreads were determined in **e**. Data are means± s.d. from three independent experiments (≥50 cells per experiment). *P<0.001; NS, not significant (unpaired *t*-test). (**f**) Fluorescence *in situ* hybridization (FISH) analysis with probes for centromeric DNA of chromosomes 3 (red), 7 (green) and 17 (blue) was performed six generations after the indicated lentiviral infection. (**g**) Quantification of the proportion of cells exhibiting centromere signals deviating from the modal in **f**. Data are means± s.d. from three independent experiments (≥350 cells for each condition per experiment). **P<0.01; NS, not significant (unpaired *t*-test) compared with control. Scale bars, 5 μm.

Finally, we investigated whether EB2 dysregulation caused chromosomal instability. To this end, we established MCF10A cells stably expressing EB2-WT and EB2-7A (Fig. 7a). Expression of EB2-7A significantly increased the number of cells with lagging chromosomes during anaphase (Fig. 7b,c). Lagging chromosomes can lead to chromosomal missegregation and aneuploidy; therefore, we next investigated aneuploidy in MCF10A cells up to 30 generations (Supplementary Fig. 8). Indeed, the incidence of aneuploidy was remarkably increased in cells expressing EB2-7A relative to that in cells expressing EB2-WT (Fig. 7d,e). Consistent with these observations, fluorescence *in situ* hybridization analysis of chromosomes 3, 7 and 17 revealed that stable expression of EB2-7A increased aneuploidy after six generations (Fig. 7f,g). These results indicated that dysregulation of EB2 phosphorylation induced chromosomal instability.

## Discussion

During mitosis, the function and localization of +TIPs, including CLIP-170, CLASP2, MCAK and EB3, are regulated by phosphorylation. Here we showed that EB2 is hyperphosphorylated at multiple sites during mitosis. Recent studies demonstrated that multi-site phosphorylation of Mal3 or Bim1p, the fission and budding yeast homologues of EB1, reduces their binding affinity to MTs^36^,^37^, and that their linker regions contribute to MT binding^25^,^56^. Our data show that multi-site phosphorylation of EB2 at the linker region connecting the CH and EBH domains, which exhibits low sequence conservation among human EBs, significantly reduced binding affinity to MTs, as in the case of the yeast EB1 homologues (Figs 2e and 4a,b). Phosphorylation at the linker region of EBs, which decreases their binding affinity for MTs, is likely to be conserved from yeasts to human; here, for the first time, we confirm this is the case for EB2.

EB2 was not phosphorylated at multiple sites during interphase, even if cells were treated with MT drugs. The timing of EB2 hyperphosphorylation was strictly regulated during mitosis (Fig. 2b–d). Our findings suggest that Aurora B and CDK1 are involved in EB2 hyperphosphorylation during mitosis; thus, the timing of EB2 phosphorylation might simply be associated with elevated activity of these mitotic kinases. Moreover, our data indicated that EB2 hyperphosphorylation was maintained in arrested metaphase cells following nocodazole block and release into MG132, suggesting that EB2 may be a downstream target of SAC, and that its phosphorylation status is maintained while the SAC is activated (and the mitotic checkpoint complex is continuously produced). Importantly, Aurora B and CDK1 contributed to maintenance of EB2 hyperphosphorylation, while the SAC remained active following washout of the SAC-activating drugs. Although Aurora B alone could decrease the MT-binding affinity of EB2, CDK1 alone was not sufficient for regulation (Fig. 3e,f). Therefore, the maintenance of EB2 hyperphosphorylation by Aurora B and CDK1 might play different roles in relation to the regulation of MT-binding affinity. The differences between the roles of Aurora B and CDK1 in the regulation of the association of EB2 with MTs are not yet clear. One possibility is that CDK1 acts as a priming kinase for Aurora B or other kinases. Interestingly, at multi-phosphorylation sites of EB2 in the linker region connecting the CH and EBH domains, consensus sequences for Aurora B and CDK1 phosphorylation are located in close proximity (Fig. 4a). In fact, CLASP2, a member of the +TIPs family, is phosphorylated by CDK1, which primes CLASP2 for association with PLK1 (ref. 15).

Aurora B has been identified as the EB2 kinase in cells (this study) and *in vitro* (this study and ref. 33). A MT-targeted EB2 mutant (EB2-Q125R) strongly localized to spindle MTs and was hyperphosphorylated in the absence of SAC-activating drugs (Fig. 4c,d). During spindle assembly in *Xenopus* egg extracts or human cells, Aurora B is localized to, and active on, spindle MTs^57^,^58^. Taken together with our result showing that multi-site phosphorylation of EB2 significantly reduced the protein's affinity to MTs, we speculate that EB2 is excluded from spindle MTs by Aurora B, to ensure proper MT dynamics and contribute to spindle re-assembly under conditions in which spindle function is compromised. Indeed, mitotic HeLa cells expressing non-phosphorylatable EB2 (EB2-7A), which can bind to polymerized MTs, exhibited a delay in chromosome alignment to the metaphase plate after monastrol washout in a manner dependent on MT binding (Fig. 5e,f). In this case, neither accumulation of monopolar spindles (Fig. 5e) nor a defect in Hec1 phosphorylation (Supplementary Fig. 7a,b) were observed; therefore, mitotic delay in EB2-7A cells is not involved in the error correction of erroneous kinetochore–MT attachments. It is important to note that non-phosphorylatable EB2 is associated with a decrease in kinetochore MT turnover, resulting in a delay in chromosome alignment to the metaphase plate and assembly of stable kinetochore–MT attachments. A recent study reported a similar stability of kinetochore–MTs induced by overexpression of the oncogene *AURKA* or by loss of the tumour suppressor gene *CHK2* (ref. 54). Consistent with our results, this stability of kinetochore–MTs does not result from interference with error correction. Furthermore, an MT-binding mutant of EB2 (EB2-K132E), which has a reduced ability to bind to MTs, can be hyperphosphorylated as efficiently as EB2-WT or a MT-targeted EB2 mutant (EB2-Q125R) (Fig. 4g). In light of recent studies showing that Aurora B substrates are phosphorylated throughout the cell during mitosis^59^, it is possible that Aurora B might phosphorylate EB2 not only on spindle MTs but also in the cytoplasm. Although Aurora B and CDK1 have been identified as the EB2 kinases, we cannot exclude the possibility that other mitotic kinases contribute to spatiotemporally regulated phosphorylation of EB2. As shown in Fig. 3e, hyperphosphorylation of EB2 was not completely abolished in the presence of inhibitors of Aurora B and CDK1 (hesperadin and roscovitine, respectively), suggesting that other kinases participate in maintaining the hyperphosphorylation of EB2.

Finally, dysfunctional temporal regulation of kinetochore MT dynamics can lead to chromosome malorientation and missegregation^5^,^54^. In fact, we showed that expression of non-phosphorylatable EB2 led to lagging chromosomes and aneuploidy (Fig. 7). Together, our findings suggest that phosphoregulation of EB2 is essential for proper mitotic progression and genomic stability. A recent study showed that EB2 overexpression is involved in perineural invasion of pancreatic cancer^60^. Further exploration of the kinases that phosphorylate EB2 and the underlying molecular mechanisms involved in regulation of mitotic EB2 will be required for an understanding of the role of EB2 dysregulation in tumour formation and progression.

## Methods

### Cell culture and materials

HeLa (JCRB), U2OS (ECACC), HCT116 (ECACC) and MCF7 (ATCC) cells were cultured in DMEM (Gibco). DLD1 (ATCC) cells were cultured in RPMI1640 (Gibco). MRC5 cells were cultured in MEM (Gibco). CHO-K1 (ATCC) cells were cultured in Ham's F-12K (Wako). All media were supplemented with 10% fetal bovine serum, penicillin (100 U ml^−1^) and streptomycin (100 μg ml^−1^). Flp-In HeLa cells (provided by Dr Patrick Meraldi, University of Geneva, Switzerland) were supplemented with zeocin (400 μg ml^−1^)^61^. MCF10A (ATCC) cells were cultured in MEBM medium (Lonza) supplemented with cholera toxin (100 ng ml^−1^). All cell lines were grown in a 5% CO_2_ atmosphere at 37 °C. Cell lines were obtained from ATCC or ECACC, as indicated above. HeLa, Flp-In HeLa and MRC5 cells were authenticated by short tandem repeat (STR) profiling and tested for mycoplasma contamination. The EB2 (*MAPRE2*) gene was amplified from HeLa complementary DNA by PCR using the forward primer 5′-CACCATGCCTGGGCCGACCCAAAC-3′ and the reverse primer 5′-GTACTCTTCCTGCTGCGGGG-3′, and then cloned into pENTR/D-TOPO (Thermo Fisher). Mutations were introduced using the QuikChange Lightning Site-Directed Mutagenesis Kit (Agilent Technologies). The small interfering RNA (siRNA)-resistant EB2-WT and mutant genes were cloned into pENTR/D-TOPO, and then transferred into the pcDNA5/FRT/TO vector (Gateway Technology, Thermo Fisher). EB2 was C-terminally tagged with 3FLAG or enhanced GFP. For establishment of cell lines stably expressing EB2, Flp-In HeLa host cells were co-transfected with a 9:1 ratio of pOG44:pcDNA5/FRT/TO expression constructs using Lipofectamine LTX (Thermo Fisher). Forty-eight hours after transfection, cell lines were selected with 200 μg ml^−1^ hygromycin B. EB2-specific siRNA was synthesized by Takara Bio. The target sequence was 5′-CCAAAUCCGAUAAAGAUUUdTdT-3′. Other siRNA sequences were as follows: Aurora A, 5′-AAGCACAAAAGCUUGUCUCCAdTdT-3′; Aurora B, 5′-AACGCGGCACUUCACAAUUGAdTdT-3′ (ref. 62); survivin, 5′-GAATTAACCCTTGGTGAATdTdT-3′ (ref. 63); and borealin (hDasra), 5′-AAAGGUCAAGCCGUGCUAACAdTdT-3′ (ref. 64). In all experiments, a duplex targeting the luciferase gene (*GL2*) was used as a control. siRNA transfections were performed using Lipofectamine RNAiMAX (Thermo Fisher) and cells were analysed 48 h after transfection. The final concentration of each reagent was as follows: nocodazole, 200 ng ml^−1^ (M1404; Sigma); paclitaxel, 10 μM (T1912; Sigma); monastrol, 100 μM (475879; Calbiochem); MG132, 20 μM (474790; Calbiochem); MLN8054, 500 nM (S1100; Selleck); hesperadin, 50 nM (375680; Calbiochem); roscovitine, 10 μg ml^−1^ (R7772; Sigma); docetaxel, 100 nM (01885; Sigma); vinblastine, 10 nM (V1377; Sigma); vincristine, 100 nM (V8879; Sigma); eribulin, 10 nM (provided by Eisai Co.); and thymidine, 2.5 mM (T1895; Sigma).

### Immunoblotting

Cells were harvested and lysed in lysis buffer (20 mM Tris pH 8.0, 150 mM NaCl, 1 mM EDTA, 0.5% NP-40, 1 mM phenylmethylsulfonyl fluoride, a protease inhibitor cocktail and a phosphatase inhibitor cocktail (Nacalai Tesque)) for 30 min on ice. Cell extracts were clarified by centrifugation. Cell lysates were boiled in SDS loading buffer. Pellet fractions were analysed as a chromatin-rich fraction. Immunoblotting was performed using the following antibodies at the indicated dilution: rat anti-EB2 (RP1) at 1:500 (sc-101490; Santa Cruz), mouse anti-EB1 at 1:500 (610534; BD Biosciences), mouse anti-cyclin A at 1:1,000 (05-374; Millipore), mouse anti-cyclin B at 1:1,000 (05-373; Millipore), mouse anti-securin at 1:2,000 (K0090-3; MBL), mouse anti-Cdc27 at 1:1,000 (610454; BD Biosciences), rabbit anti-phospho-histone H3 (S10) at 1:2,000 (3377; Cell Signaling Technology), mouse anti-histone H3 at 1:1,000 (3638; Cell Signaling Technology), mouse anti-α-tubulin at 1:2,000 (T6199; Sigma), mouse MPM-2 at 1:500 (05-368; Millipore), rabbit anti-phospho-Aurora A (T288)/phospho-Aurora B (T232)/phospho-Aurora C (T198) at 1:2,000 (2914; Cell Signaling Technology), mouse anti-Aurora A at 1:500 (NCL-L-AK2; Leica Biosystems), rabbit anti-Aurora B at 1:500 (3094; Cell Signaling Technology), rabbit anti-survivin at 1:1,000 (NB500-201; Novus Biologicals), mouse anti-borealin at 1:1,000 (provided by Dr William C. Earnshaw, University of Edinburgh, UK), mouse anti-β-actin at 1:5,000 (A5316; Sigma), mouse anti-DYKDDDDK (FLAG) at 1:1,000 (018-22381; Wako), mouse anti-HA at 1:1,000 (M185-3; MBL) and rabbit anti-GFP at 1:1,000 (632593; Clontech Takara Bio). Quantitative analysis was performed using the ImageQuant TL software (GE Healthcare). Images have been cropped for presentation. Uncropped images of immunoblotting shown in Figs 1, 2, 3, 4, 5 and 7 are included in Supplementary Fig. 10.

### Immunofluorescence

For staining of EB1 and EB2, cells were rinsed in PBS at 37 °C, fixed in cold methanol for 3 min at −20 °C, blocked (PBS containing 2% BSA and 2% normal goat serum) for 30 min at room temperature and incubated with the following antibodies at the indicated dilution: rat anti-EB2 (RP1) at 1:50 (sc-101490; Santa Cruz), mouse anti-EB1 at 1:100 (610534; BD Biosciences) and rabbit anti-DDDK-tag at 1:1,000 (PM020 MBL). For double staining of tubulin and centromere, cells were rinsed in PBS at 37 °C, fixed in 4% paraformaldehyde for 15 min at 37 °C, permeabilized (PBS containing 0.1% Triton X-100) for 5 min at room temperature, blocked (PBS containing 2% BSA and 2% normal goat serum) for 30 min at room temperature and incubated with the following antibodies at the indicated dilution: mouse anti-α-tubulin at 1:2,000 (T6199; Sigma) and human anti-centromere (ACA) at 1:10,000 (HCT-0100; Immunovision). For phospho-Hec1 staining, cells were prepared as previously described^50^. Briefly, cells were rinsed in PHEM buffer (60 mM PIPES, 25 mM HEPES, 10 mM EGTA and 4 mM MgSO_4_ pH 6.9) at 37 °C, pre-extracted (PHEM buffer containing 1% Triton X-100 and 500 nM okadaic acid) for 5 min at 37 °C, fixed in 3.7% paraformaldehyde for 20 min at 37 °C, blocked (PHEM buffer containing 10% boiled donkey serum) for 1 h at room temperature and incubated with the following antibodies at the indicated dilution: rabbit anti-phosphorylated Ser55-Hec1 at 1:2,000 (provided by Dr Jennifer G. DeLuca, Colorado State University, USA) and mouse anti-Hec1 at 1:2,000 (ab3613; Abcam). Secondary antibodies conjugated to Alexa Fluor 488, 555 or 647 (Molecular Probes) were used at 1:2,000 dilution. After a wash in PBS containing 4,6-diamidino-2-phenylindole (DAPI) for 5 min, the coverslips were mounted in ProLong Gold (Thermo Fisher).

### Image acquisition and analysis

For fixed-cell experiments, fluorescence image acquisition was performed using a Nikon A1R confocal imaging system controlled by the Nikon NIS Elements software (Nikon). The objective lens was an oil immersion Plan-Apo × 60 numerical aperture (NA) 1.40 lens (Nikon). Images were acquired as Z-stacks at 0.2-μm intervals and maximum-intensity projections were generated using the NIS Elements software (Nikon). Kinetochore fluorescence intensity analysis and EB2 fluorescence intensity analysis were performed using the MetaMorph software (Molecular Devices). For kinetochore fluorescence intensity analysis, all Z-stack sections containing the fluorescence signal were background-subtracted and summed, and a region of interest was defined for each kinetochore. Hec1 and pHec1 intensities were expressed relative to the intensity of ACA. For EB2 fluorescence intensity analysis, a region of interest in an individual cell was defined using the maximum-intensity projection image of MTs. All *Z*-stack sections containing the fluorescence signal were summed. EB2-FLAG intensity was expressed relative to the intensity of tubulin.

For live-cell imaging of mitotic duration, HeLa cell lines stably expressing either RNAi-resistant EB2-WT or the non-phosphorylatable mutant (EB2-7A) were transfected with EB2-targeting siRNAs, and 24 h later with plasmids harbouring histone H2B-mCherry. The cells were imaged in 35-mm glass bottom dishes (IWAKI) containing phenol red-free DMEM (Gibco). The imaging medium was maintained at 37 °C under 5% CO_2_ in a stage-top incubator (Tokai Hit). Images were acquired every 5 min for 24 h with a 20-ms exposure time using a Plan-Apo × 20 NA 0.75 lens (Nikon) objective on an inverted fluorescence microscope (Nikon Eclipse Ti-E) equipped with an ORCA-Flash 4.0 V2 camera (Hamamatsu Photonics). The interval from NEBD to the onset of anaphase was calculated manually.

### *In vitro* kinase assay

EB2 proteins tagged with His at the C terminus were expressed in Rosetta2 (DE3) pLysS and purified with MagneHis Ni-Particles (Promega). Purified EB2, histone H1 (as a positive control of CDK1, New England Biolabs) and histone H3 (as a positive control of Aurora B, New England Biolabs) proteins were incubated with active CDK1-Cyclin B (Millipore) and/or active Aurora B (Abcam) in kinase buffer. *In vitro* kinase assays were carried out in 30 μl of reaction buffer (50 mM Tris-HCl, 10 mM MgCl_2_, 1 mM EGTA, 2 mM dithiothreitol, 0.01% Brij 35, 50 μM cold ATP, 5 μCi [γ-^32^P]-ATP and 1 μg of purified EB2 proteins pH 7.5) containing 50 ng of CDK1-Cyclin B or 50 ng of Aurora B. Reactions were incubated at 30 °C for 30 min. Reaction samples were terminated by adding 4 × Laemmli buffer, boiled and subjected to SDS–PAGE followed by autoradiography.

### MT co-sedimentation assay

MT co-sedimentation assays were performed as previously described^65^,^66^,^67^. Briefly, HeLa cells were lysed in PEM buffer (80 mM PIPES, 1 mM EGTA and 1 mM MgCl_2_ pH 6.8) supplemented with a protease inhibitor cocktail and a phosphatase inhibitor cocktail (Nacalai Tesque). Cell lysates were centrifuged at 16,000 *g* for 15 min at 4 °C and the high-speed supernatant was isolated. Porcine brain tubulin (Cytoskeleton, Inc.) was added to warm PEM buffer containing high-speed supernatant, 1 mM GTP and 20 μM taxol, and MT polymerization was carried out for 30 min at 37 °C. Alternatively, 20 μM colchicine was added instead of taxol and the solution was maintained on ice for 30 min, to prevent MT polymerization. Samples were centrifuged at 100,000 *g* for 35 min at room temperature. The WP and cold pellet (CP) were separated from the WS and cold supernatant. The pellet samples were resuspended in PEM buffer, in a volume equal to that of the supernatant.

### Monastrol washout experiment

For fixed-cell experiments, Flp-In HeLa parental cells or cell lines stably expressing either RNAi-resistant EB2-WT or mutants were transfected with GL2- or EB2-targeting siRNAs. After 48 h, cells were treated with monastrol for 2 h, transferred to fresh medium containing MG132 for 1 h and then fixed and co-stained with an anti-tubulin antibody, ACA and DAPI.

For live-cell imaging, cells stably expressing histone H2B-AcGFP were co-transfected with plasmids harbouring pericentrin-RFP^68^ (provided by Dr Linda Wordeman, University of Washington, USA) and siRNAs. After monastrol washout, images were acquired every 1 min for 90 min with an exposure time of 20 ms (for GFP) or 50 ms (for RFP) using an oil-immersion Plan-Apo × 60/1.4 NA DIC lens objective (Nikon) on an inverted fluorescence microscope (Nikon Eclipse Ti-E) equipped with an ORCA-Flash 4.0 V2 camera (Hamamatsu Photonics). Images were acquired as *Z*-stacks at 1.2-μm intervals and maximum-intensity projections were generated using the NIS Elements software (Nikon). The intervals from monastrol release to spindle bipolarization and to chromosome alignment were calculated manually.

### Measurements of the half-life of kinetochore MT turnover

For photoactivation studies, Flp-In HeLa cells stably expressing both photoactivatable GFP–α-tubulin and RNAi-resistant EB2 (WT or non-phosphorylatable mutant) were transfected with EB2-targeting siRNAs for 48 h. Late prometaphase cells were photoactivated on one side of the bipolar spindle with a 405-nm laser on a Nikon A1R confocal imaging system. Images were acquired every 10 s for the first two images and every 20 s thereafter. The intensities of the fluorescent bar were measured as described by DeLuca *et al.*^51^, in which the fluorescence value of a corresponding background region in the opposite half of the spindle was subtracted from the value of the activated bar region. The values were corrected for photobleaching by determining the amount of fluorescence loss of activated taxol-treated spindles over time. The average decrease in fluorescence due to photobleaching was collected for multiple cells (*n*=10) at each time point. Fluorescence values were normalized to the first time point after photoactivation for each cell and the average intensity at each time point was fitted to a two-phase exponential decay curve using the programme PRISM (GraphPad Software), in which the normalized fluorescence (*y*) is described by: *y*=*A*_1_exp (–*k*_*1*_*t*)+*A*_2_exp (–*k*_*2*_*t*). *A*_1_ represents the rapid turnover of non-kinetochore MTs and *A*_2_ represents the contribution of stable kinetochore MTs, *k*_*1*_ and *k*_*2*_ are their respective rate constants of turnover and *t* is the time after photoactivation. The turnover half-life was calculated as ln 2/*k*.

### Cold-induced MT depolymerization assay

Flp-In HeLa parental cells or cell lines stably expressing either RNAi-resistant EB2-WT or mutants were transfected with GL2- or EB2-targeting siRNAs. After 48 h, cells were incubated in an ice-water bath for 15 min and then fixed and co-stained with an anti-tubulin antibody, ACA and DAPI. For quantification, 25 images were acquired as *Z*-stacks at 0.2-μm intervals and summed. The intensities of the fluorescence were measured as described by DeLuca *et al.*^69^ Briefly, a circle that included the whole spindle was chosen as the area for quantification for all conditions. A background circle, which was 1.25-fold larger than the original circle, was used to measure the background fluorescence. MT intensity was expressed relative to the intensity of ACA.

### Chromosome spreads

Chromosome spreading was performed by GTG (G-bands by trypsin using Giemsa). Cells were treated with 20 ng ml^−1^ colchicine for 1 h, collected and hypotonically swollen in 75 mM KCl for 20 min at 37 °C. Cells were fixed in Carnoy's fixative solution (75% methanol and 25% acetic acid) with several changes of the fixative. Cells were dropped onto cooled glass slides and dried at room temperature. Chromosomes were trypsinized and stained in 5% Giemsa for 10 min, rinsed with PBS, air dried and mounted.

### Lentiviral infections

EB2-WT and mutant genes cloned into pENTR/D-TOPO and pENTR5'/EF1αp were co-transferred into the pcLenti6.4/R4R2/V5-DEST vector (Gateway Technology, Thermo Fisher). EB2 was C-terminally FLAG-tagged. Lentiviral stocks were produced using the ViraPower Lentiviral Expression System (Thermo Fisher). MCF10A cells were infected, to generate a polyclonal population expressing FLAG-tagged EB2.

### Fluorescence *in situ* hybridization

Fluorescence *in situ* hybridization analyses were performed as previously described^70^ using ZytoLight SPEC p16/CEN3/7/17 Quadruple Color Probes (Zytovision). Briefly, cells on coverslips were fixed in Carnoy's fixative solution (75% methanol and 25% acetic acid), washed in 2 × SSC (1 × SSC is 0.15 M NaCl and 0.015 M sodium citrate) for 2 min and then dehydrated in an ethanol series (70, 85 and 100%; 2 min each following air drying). The DNA probes were pre-warmed at 37 °C, placed onto the coverslips and denatured at 75 °C for 2 min. The coverslips were then incubated overnight at 37 °C, washed with 4 × SSC containing 0.05% Tween 20 for 5 min and then incubated in 0.25 × SSC at 72 °C for 2 min. After a wash in 4 × SSC containing 0.05% Tween 20 for 30 s, the coverslips were mounted in ProLong Diamond containing DAPI (Thermo Fisher).

### Statistical analyses

The data were analysed using the Student's *t*-test or the Mann–Whitney *U*-test. We tested data for normality and variance between the groups being statistically compared. Statistical calculations were carried out using the programme PRISM (GraphPad Software). All immunoblotting and immunofluorescence experiments were repeated at least twice.

### Accession numbers and multiple sequence alignment

Multiple protein sequence alignments were carried using the programme CLUSTALW, DDBJ (http://clustalw.ddbj.nig.ac.jp/top-j.html) using the full amino-acid sequences of EB1 (NP_036457), EB2 (NP_055083) and EB3 (NP_001007657).

## Additional information

**How to cite this article:** Iimori, M. *et al.* Phosphorylation of EB2 by Aurora B and CDK1 ensures mitotic progression and genome stability. *Nat. Commun.* 7:11117 doi: 10.1038/ncomms11117 (2016).

## Supplementary Material

Supplementary Information Supplementary Figures 1-10

Supplementary Movie 1 An example of Flp-In HeLa cells after monastrol washout. Flp-In HeLa cells stably expressing histone H2B-AcGFP were co-transfected with plasmids harbouring pericentrin-RFP and siRNAs. Time lapse was acquired using a 60× /1.4 NA DIC lens objective, with a stack taken every 1 minute. The movie is presented as a maximum intensity projection. The movie is played at 6 frames per second. Scale bar, 10 μm.
