# Supplementary material for: Phosphorylation of EB2 by Aurora B and CDK1 ensures mitotic progression and genome stability
Source: Nat Commun. 2016 Mar 31;7:11117. doi: 10.1038/ncomms11117 (PMC4821873; doi:10.1038/ncomms11117)
Supplement: Supplementary Information — Supplementary Figures 1-10 [file ncomms11117-s1.pdf]

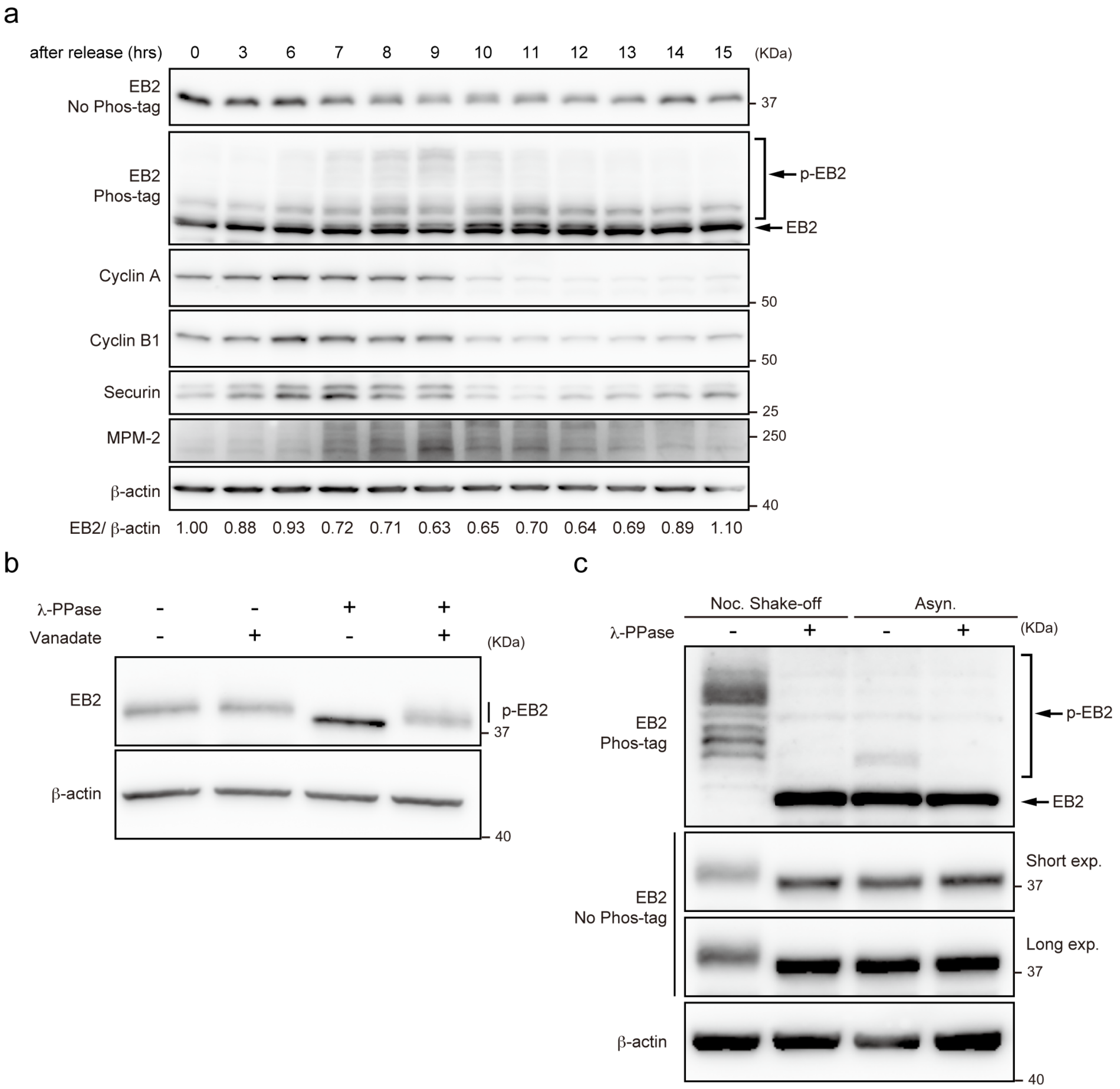

Supplementary Figure 1. EB2 is phosphorylated during mitosis.
(**a**) HeLa cells were synchronized by a double-thymidine block and release into fresh medium (see Supplementary Fig. 9a for experimental scheme). Immunoblot analysis was carried out using antibodies against the indicated proteins; SDS-PAGE was performed using the Phos-tag gel for EB2 detection. Relative EB2 protein levels were quantified. (**b**) Phosphatase assay of hyper-shifted EB2. Lysates of nocodazole-arrested cells were treated with or without 100 units of lambda phosphatase (New England Biolabs). As a phosphatase inhibitor, 1 mM sodium orthovanadate was added. The reaction was incubated for 30 minutes at 30°C, and immunoblot analysis was carried out using the anti-EB2 antibody. (**c**) Phosphatase assay of shifted EB2 in asynchronous (Asyn.) or nocodazole-arrested (Noc.) extracts of whole or shake-off HeLa cells. Note that Phos-tag gels were used only in Supplementary Fig. 1a and 1c in this study.

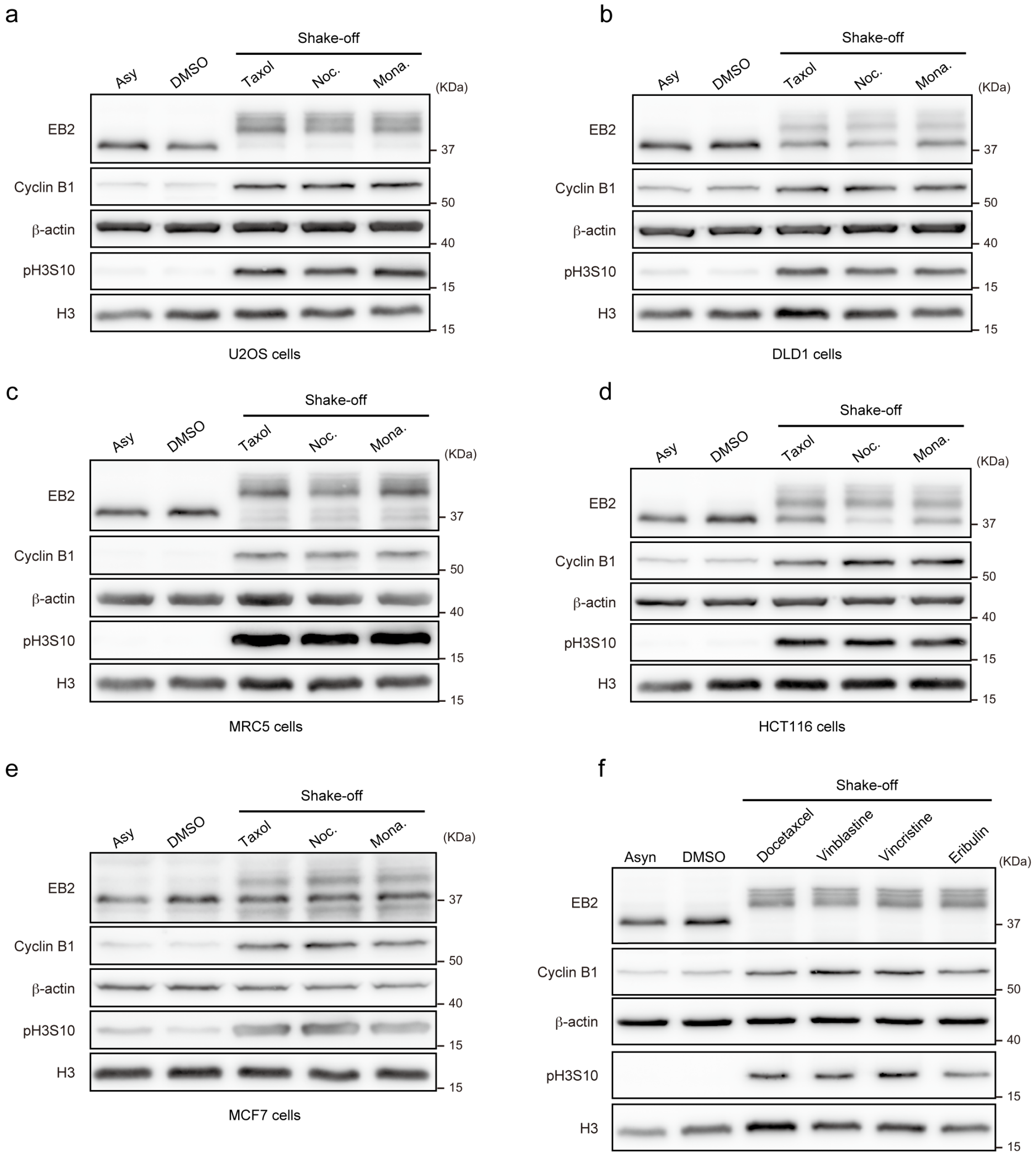

Supplementary Figure 2. Hyper-phosphorylation of EB2 is observed in several cell lines.

(**a**–**e**) Immunoblot analysis of EB2 in asynchronous (Asyn.), taxol-arrested, nocodazole-arrested (Noc.), and monastrol-arrested (Mona.) extracts from mitotic (shake-off) cells. (**f**) Immunoblot analysis of EB2 in mitotic (shake-off) HeLa cells arrested with anti-cancer drugs.

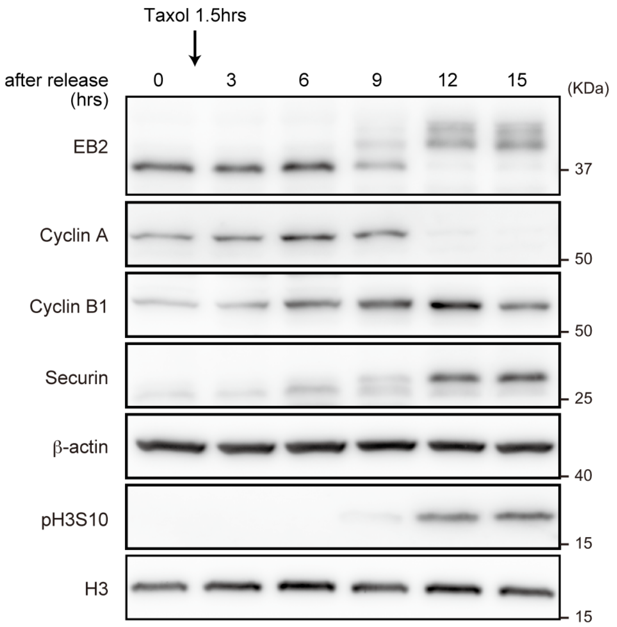

Supplementary Figure 3. Hyper-phosphorylation of EB2 by taxol treatment is consistent with mitotic entry.

HeLa cells were synchronized by a double-thymidine block, and taxol was added 1.5 h after release (see Supplementary Fig. 9b for experimental scheme). Immunoblot analysis was carried out using antibodies against the indicated proteins.

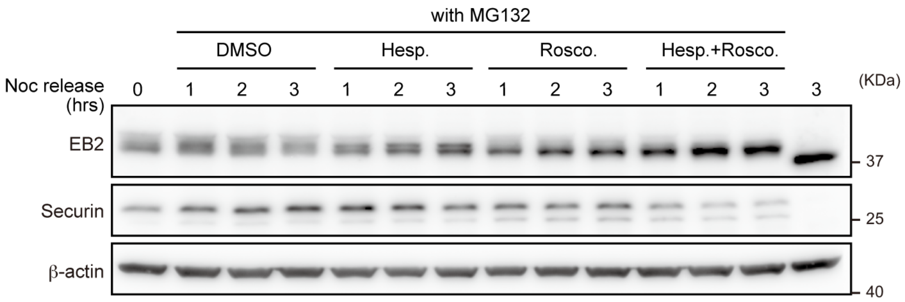

Supplementary Figure 4. Inhibition of Aurora B or CDK1 partially abolishes the hyper-phosphorylation of EB2.

HeLa cells were synchronized by a single thymidine block, and then after 3 h released into nocodazole for 11 h. Synchronized mitotic HeLa cells were harvested by mitotic shake-off, washed, and released into MG132 with or without roscovitine (CDK1 inhibitor) and/or hesperadin (see Supplementary Fig. 9f for experimental scheme).

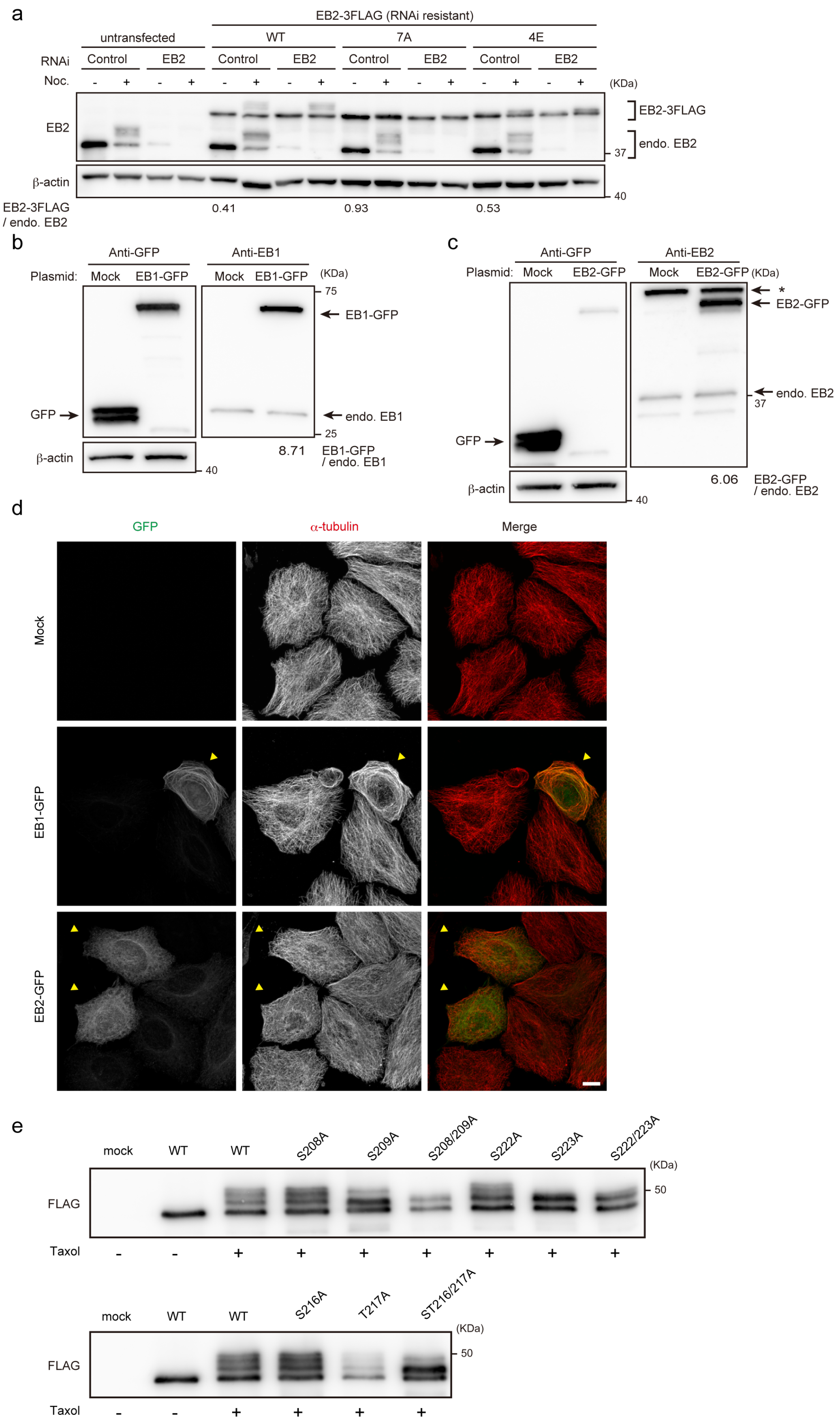
a
EB2-3FLAG (RNAi resistant)
untransfected
WT
7A
4E
RNAi
Control
EB2
Noc.
(KDa)
EB2
EB2-3FLAG
endo. EB2
37
β-actin
40
EB2-3FLAG / endo. EB2
0.41
0.93
0.53
b
Anti-GFP
Anti-EB1
Plasmid:
Mock
EB1-GFP
75
EB1-GFP
GFP
endo. EB1
25
β-actin
40
8.71
EB1-GFP / endo. EB1
c
Anti-GFP
Anti-EB2
Mock
EB2-GFP
*
EB2-GFP
endo. EB2
37
GFP
β-actin
40
6.06
EB2-GFP / endo. EB2
d
GFP
α-tubulin
Merge
Mock
EB1-GFP
EB2-GFP
e
mock
WT
S208A
S209A
S208/209A
S222A
S223A
S222/223A
(KDa)
50
FLAG
Taxol
S216A
T217A
ST216/217A

Supplementary Figure 5. Establishment of stable EB2 cell lines.
(**a**) HeLa Flp-In cell lines were established that stably expressed RNAi-resistant EB2-FLAG wild-type (EB2-WT), non-phosphorylatable mutant (EB2-7A), or phospho-mimetic EB2 (EB2-4E). Noc., nocodazole. The EB2-FLAG/endogenous EB2 protein level ratio was quantified. (**b**–**d**) Ectopic expression of EB1-GFP (**b**) or EB2-GFP (**c**). The EBs-GFP/endogenous EBs protein level ratios were quantified. Representative images of EBs-GFP-expressing cells are indicated by yellow arrowheads in **d**. The asterisk indicates a nonspecific cross-reacted protein band recognized by the anti-EB2 antibody. Scale bars, 10 μm. (**e**) Cell extracts from HeLa cells transiently expressing EB2-FLAG wild-type or its phosphomutants in the presence of taxol were immunoblotted with an anti-FLAG antibody.

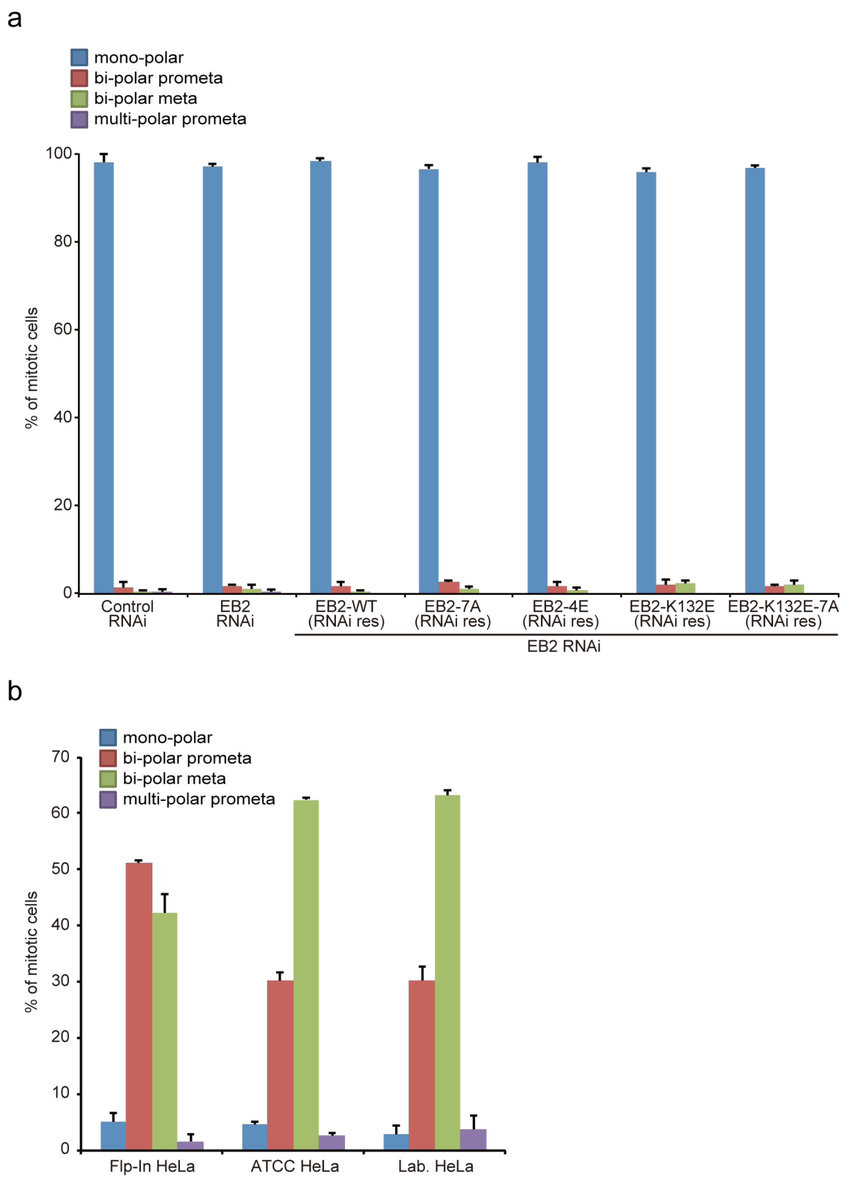

Supplementary Figure 6. Monastrol washout experiment.

(**a**) HeLa Flp-In cells expressing RNAi-resistant EB2-FLAG wild-type (EB2-WT), its non-phosphorylatable mutant (EB2-7A), phospho-mimetic EB2 (EB2-4E), a MT-binding site mutant (EB2-K132E), or its non-phosphorylatable mutant (EB2-K132E-7A) were transfected with siRNA against endogenous EB2 and incubated for 48 h. Cells were treated with monastrol for 2 h, and then fixed and co-stained with an anti-tubulin antibody, ACA, and DAPI. The proportion of early mitotic cells in the indicated four categories is shown in Fig. 5d. Data are means ± s.d. from three independent experiments (≥ 250 cells per experiment). (**b**) Different HeLa cell lines were treated with monastrol for 2 h, transferred into fresh medium containing MG132 for 1 h, and then fixed and co-stained with an anti-tubulin antibody, ACA, and DAPI. The proportion of early mitotic cells in the indicated four categories is shown in Fig. 5d. Data are means± s.d. from three independent experiments (≥ 250 cells per experiment).

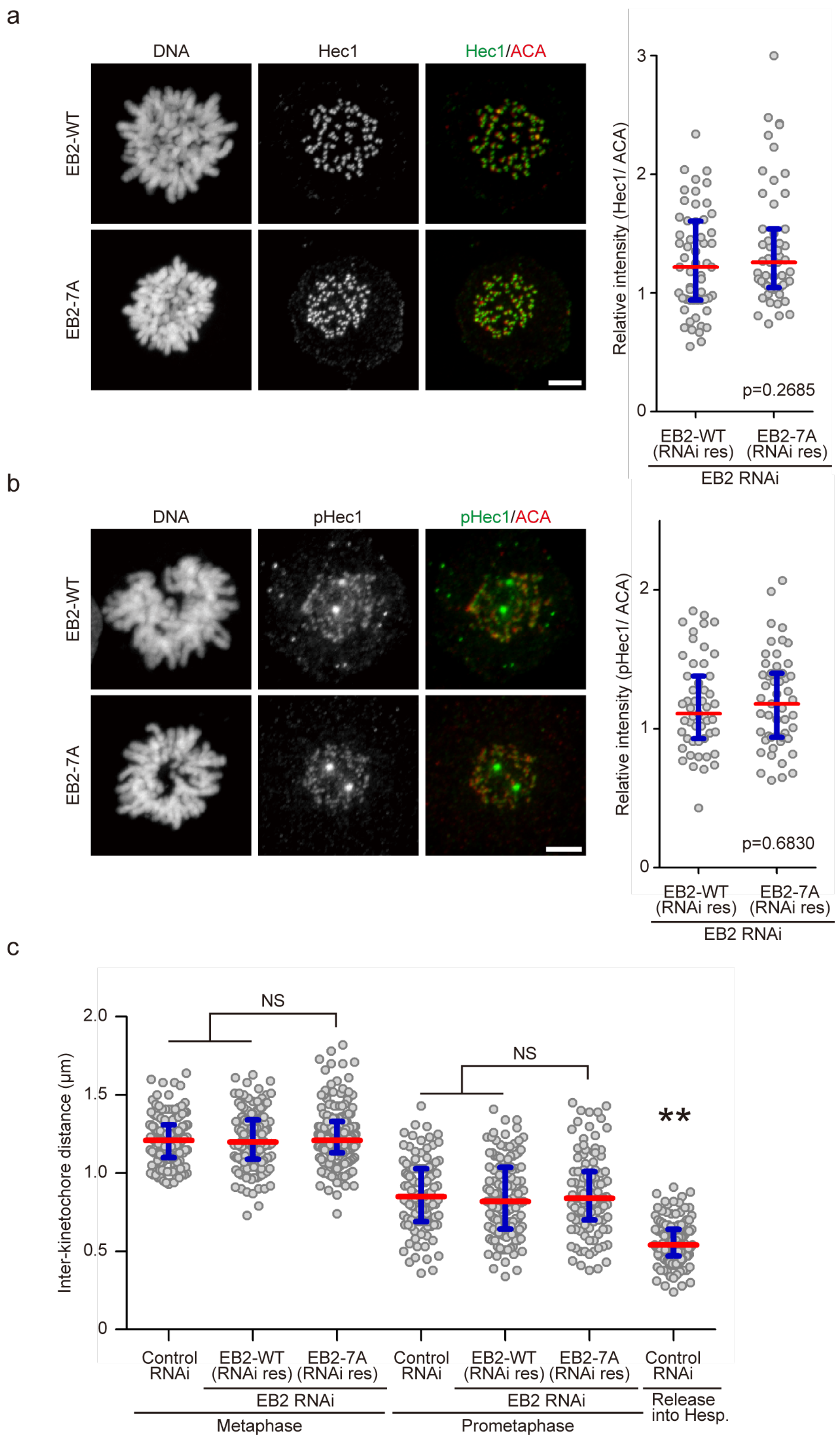

Supplementary Figure 7. Hec1 phosphorylation at kinetochores and interkinetochore distances.

(**a, b**) HeLa Flp-In cells expressing RNAi-resistant EB2-FLAG wild-type (EB2-WT) or the non-phosphorylatable mutant (EB2-7A) were immunostained with anti-Hec1 (**a, left)**

and anti–phospho-Hec1 (pSer44-Hec1; **b,** left) antibodies, and kinetochore fluorescence intensities were quantitated (**a, b,** right**)**. Intensities were normalized relative to a general kinetochore marker (ACA). Open circles represent individual kinetochores. Red lines, medians; blue bars, inter-quartile range. For each experiment, n ≥ 50 kinetochore pairs were measured from at least 10 cells. *P* values are represented on the graphs (Mann-Whitney *U*-test). Scale bars, 5 μm. (**c**) Quantification of interkinetochore distances after monastrol washout in HeLa Flp-In cells expressing RNAi-resistant EB2-FLAG wild-type (EB2-WT) or EB2 mutants. The *n* values are as follows: control metaphase, *n* = 157 kinetochore pairs, 13 cells; EB2-WT metaphase, *n* = 161 kinetochore pairs, 14 cells; EB2-7A metaphase, *n* = 185 kinetochore pairs, 15 cells; control prometaphase, *n* = 111 kinetochore pairs, 12 cells; EB2-WT prometaphase, *n* = 128 kinetochore pairs, 12 cells; EB2-7A prometaphase, *n* = 112 kinetochore pairs, 11 cells; and cells released into hesperadin, *n* = 112 kinetochore pairs, 11 cells. ***P* < 0.001, NS, not significant (Mann-Whitney *U*-test).

a

chromosome number : 47 48 49

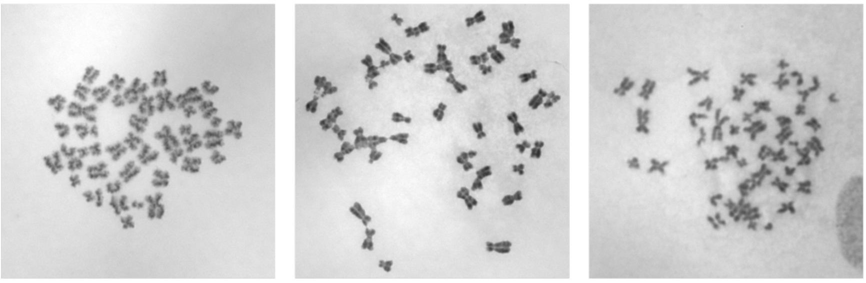

b

47,XX,i(1)(q10),add(3)(p11),add(8)(p11.2),add(9)(p13),
+mar1[1]

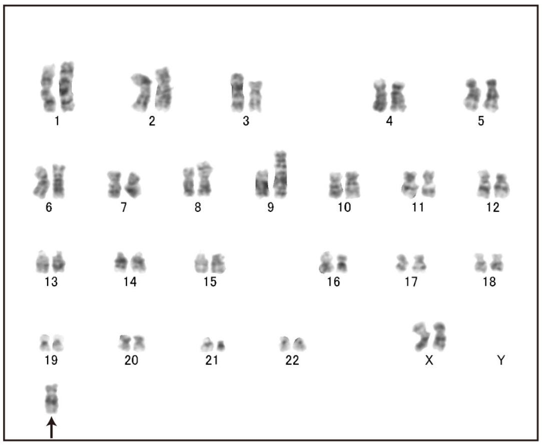

48,idem,+20[7]

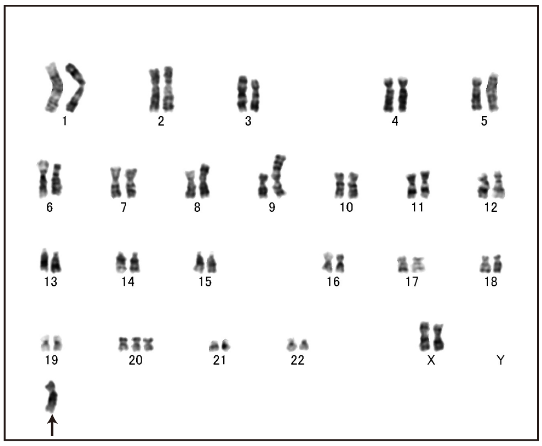

49,idem,+i(1),+20[1]/

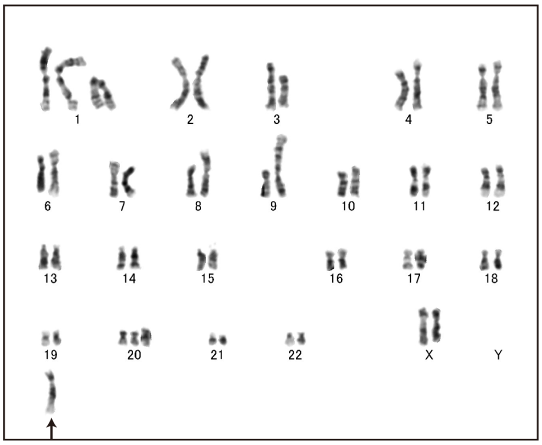

49,idem,+15,+20[1]

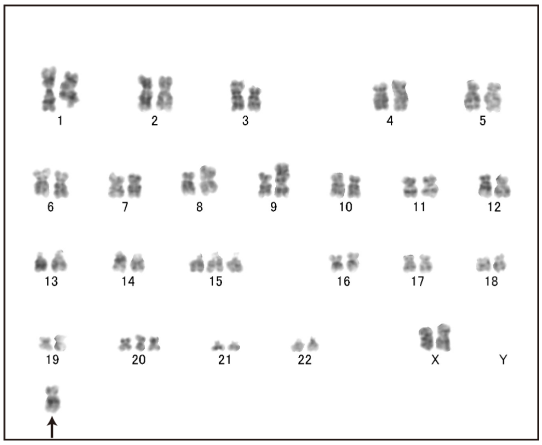

Supplementary Figure 8. Karyotype of MCF10A cells expressing non-phosphorylatable EB2.

(**a**) Examples of chromosome spreads exhibiting karyotypes deviating from the modal chromosome number. The modal chromosome number is 48 in MCF10A cells. (**b**) Examples of aneuploidy detected by G-banded karyotyping. Arrows indicate the marker chromosome.

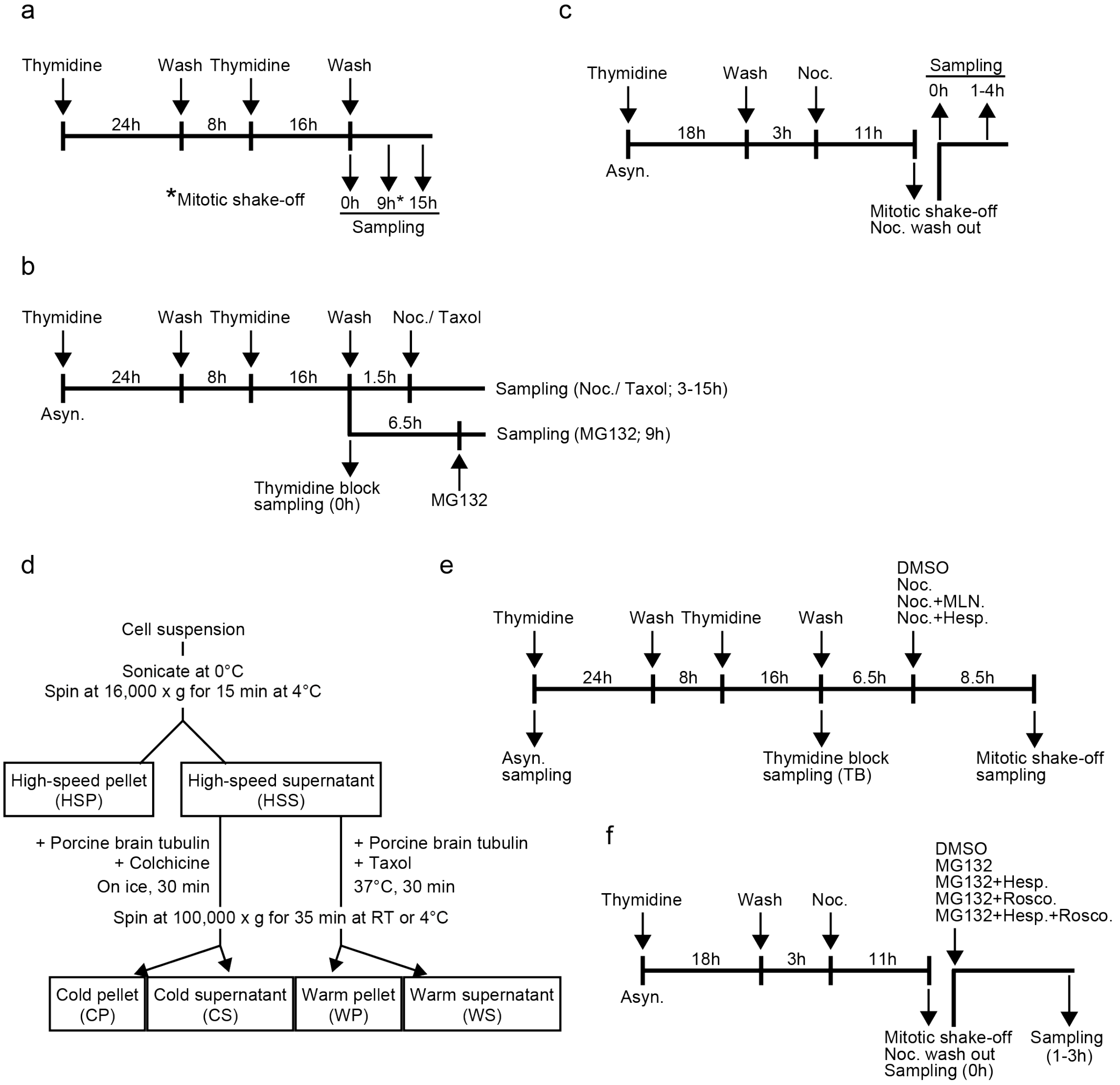

Supplementary Figure 9. Experimental schemes.
Schemes of experiments shown in Fig. 1b (**a**), Fig. 2b–c (**b**), Fig. 2d (**c**), Fig. 2e and 3e (**d**), Fig. 3b (**e**), and Fig. 3e (**f**). Asyn, asynchronous; Noc., nocodazole; MLN, MLN8054; Hesp., hesperadin; Rosco., roscovitine.

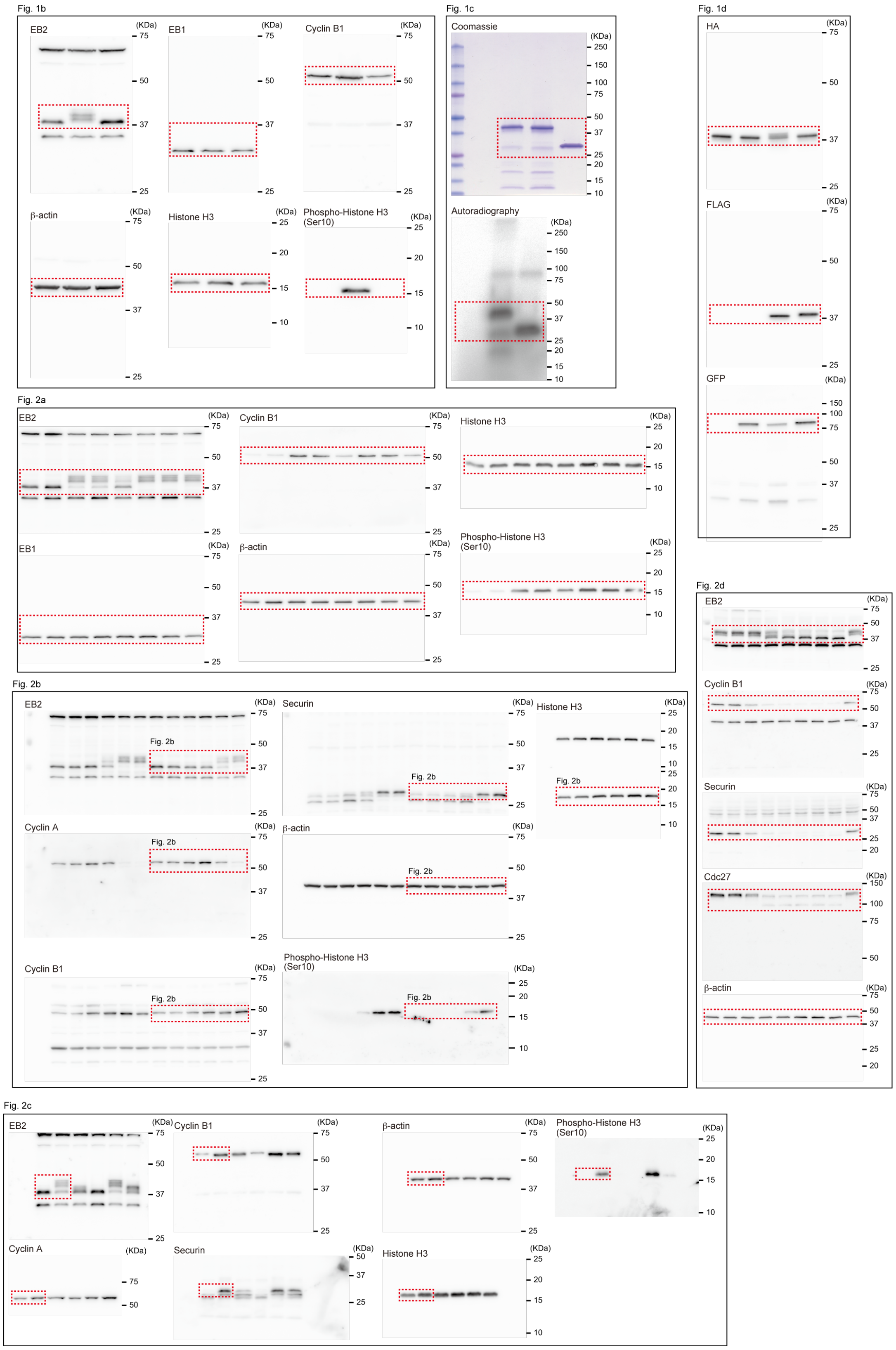

Supplementary Figure 10. Uncropped images of immunoblot. Red boxes show cropped regions.

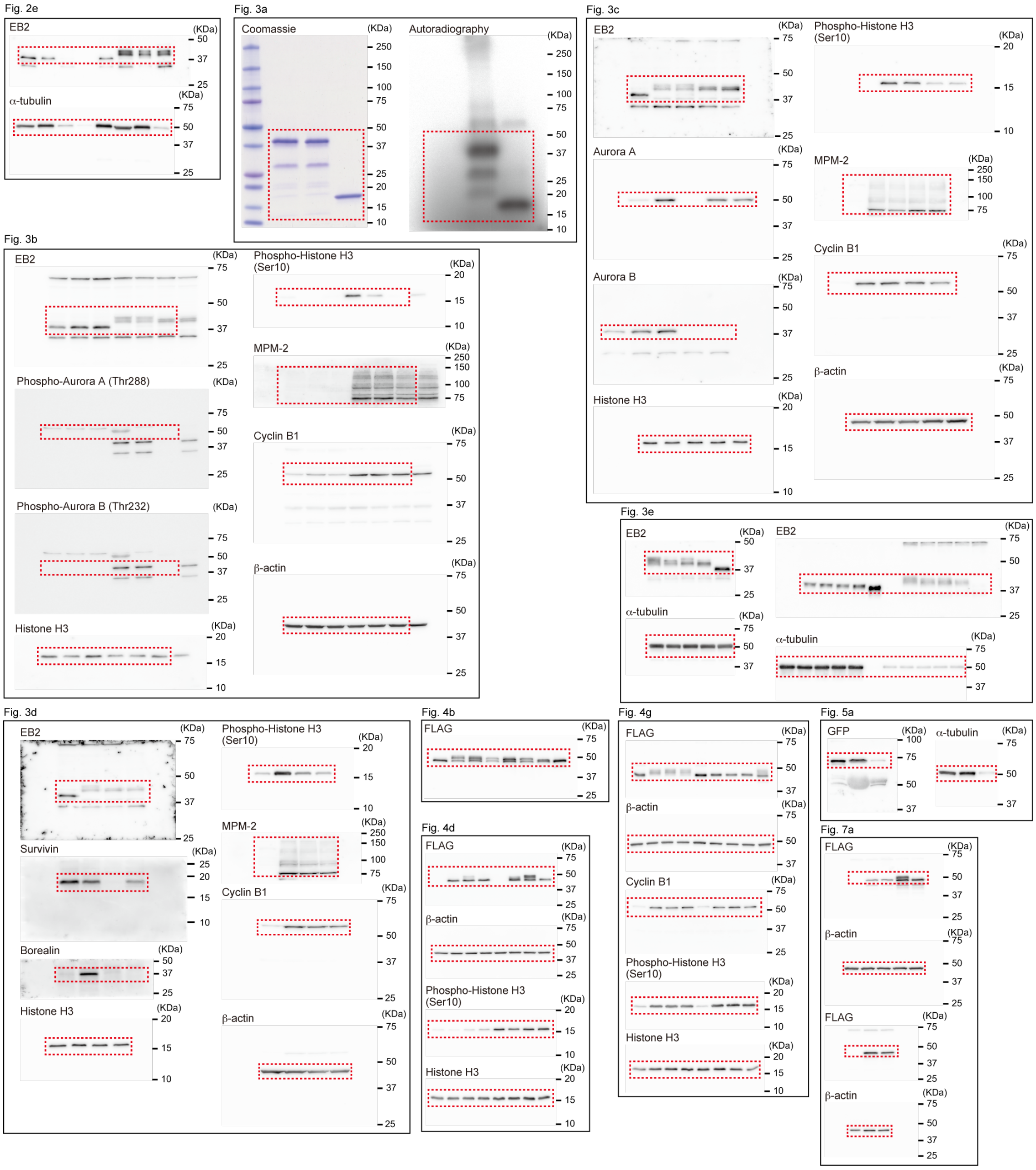

Supplementary Figure 10. Continued.
